# Diversity and disparity through time in the adaptive radiation of Antarctic notothenioid fishes

**DOI:** 10.1111/jeb.12570

**Published:** 2015-01-30

**Authors:** M Colombo, M Damerau, R Hanel, W Salzburger, M Matschiner

**Affiliations:** * Zoological Institute, University of BaselBasel, Switzerland; † Thünen Institute of Fisheries EcologyHamburg, Germany; ‡ Centre for Ecological and Evolutionary Synthesis (CEES), Department of Biosciences, University of OsloOslo, Norway

**Keywords:** adaptive radiation, early burst, geometric morphometrics, incomplete lineage sorting, species tree

## Abstract

According to theory, adaptive radiation is triggered by ecological opportunity that can arise through the colonization of new habitats, the extinction of antagonists or the origin of key innovations. In the course of an adaptive radiation, diversification and morphological evolution are expected to slow down after an initial phase of rapid adaptation to vacant ecological niches, followed by speciation. Such ‘early bursts’ of diversification are thought to occur because niche space becomes increasingly filled over time. The diversification of Antarctic notothenioid fishes into over 120 species has become one of the prime examples of adaptive radiation in the marine realm and has likely been triggered by an evolutionary key innovation in the form of the emergence of antifreeze glycoproteins. Here, we test, using a novel time-calibrated phylogeny of 49 species and five traits that characterize notothenioid body size and shape as well as buoyancy adaptations and habitat preferences, whether the notothenioid adaptive radiation is compatible with an early burst scenario. Extensive Bayesian model comparison shows that phylogenetic age estimates are highly dependent on model choice and that models with unlinked gene trees are generally better supported and result in younger age estimates. We find strong evidence for elevated diversification rates in Antarctic notothenioids compared to outgroups, yet no sign of rate heterogeneity in the course of the radiation, except that the notothenioid family Artedidraconidae appears to show secondarily elevated diversification rates. We further observe an early burst in trophic morphology, suggesting that the notothenioid radiation proceeds in stages similar to other prominent examples of adaptive radiation.

## Introduction

Adaptive radiation, that is the evolution of a multitude of species as a consequence of the adaptation to new ecological niches, is considered to be responsible for much of the diversity of life on Earth (Simpson, 1953; Schluter, 2000). In general, adaptive radiation is thought to result from ecological opportunity in the form of vacant ecological niches that may have become available due to the colonization of new habitats, the extinction of antagonists or the emergence of evolutionary key innovations that allow the invasion of new adaptive zones (Heard & Hauser, 1995; Schluter, 2000; Yoder *et al*., 2010). Starting from a single ancestor, adaptively radiating groups are expected to differentiate into an array of morphologically and ecologically diverse species filling multiple available ecological niches. Mathematical models of adaptive radiation predict that rates of diversification and morphological evolution are inversely correlated with available niche space, thus leading to a slowdown in diversification rates as niche space becomes increasingly filled (Gavrilets & Vose, 2005; Gavrilets & Losos, 2009). As a result, both speciation events and morphological change would be concentrated in an ‘early burst’ near the beginning of the history of an adaptive radiation (Simpson, 1953; Erwin, 1994; Losos & Miles, 2002).

Temporally declining rates of speciation are often observed in molecular phylogenies of radiating clades, including Anolis lizards of the Caribbean islands (Rabosky & Glor, 2010), North American wood warblers (Rabosky & Lovette, 2008), squamates (Burbrink *et al*., 2012), Neotropical cichlid fishes (López-Fernández *et al*., 2013) and bats (Yu *et al*., 2014). In addition, inverse relationships between radiation age and species counts have been found in the replicate adaptive radiations of *Tetragnatha* spiders on the Hawaiian islands (Gillespie, 2004), and in those of cichlid fishes in East African Rift lakes (Seehausen, 2006). This suggests that not only speciation rates, but also the total number of species can decline subsequently to an early burst, a phenomenon termed ‘overshooting’ (Gavrilets & Losos, 2009).

However, most empirical support for early bursts in morphological disparity derives from paleontological studies, which show that fossil groups often obtain maximum disparity early in their history, followed by subsequent decline (Foote, 1997). Several methods have been developed to infer early bursts in disparity from extant species on the basis of phylogenetic analyses (Harmon *et al*., 2003, 2010; Slater & Pennell, 2014). In practice, however, these methods often fail to detect early bursts in morphological diversification in even the most prominent examples of adaptive radiation (Harmon *et al*., 2010; but see Mahler *et al*., 2010; Slater *et al*., 2010; López-Fernández *et al*., 2013).

Adaptive radiation has also been proposed to progress in stages in the sense that diversification occurs along different axes at different intervals of a radiation (Streelman & Danley, 2003; Ackerly *et al*., 2006; Gavrilets & Losos, 2009). Verbal models, as well as a mathematical theory of speciation (Gavrilets, 2004), predict that diversification would (i) be driven by divergence according to macrohabitat, followed by (ii) increasing divergence with respect to microhabitat, (iii) traits that control both for local adaptation and nonrandom mating and (iv) traits that control for survival and reproduction. However, the order of stages seems to depend on diverse factors that differ between adaptive radiations. For example, *Phylloscopus* leaf warblers apparently diverged in the order of body size, foraging morphology/behaviour and then habitat (Richman, 1996), and trophic morphology has been suggested to diversify before macrohabitat adaptations in cichlids from Lake Tanganyika (Muschick *et al*., 2014). In contrast, Lake Malawi cichlids and marine parrotfish were found to diverge first according to habitat, followed by trophic morphology and sexually selected traits (Streelman & Danley, 2003).

The diversification of Antarctic notothenioid fishes into over 120 extant species represents a prime example of an adaptive radiation in a marine environment. Notothenioids dominate the waters surrounding the Antarctic continent both by species number (47%) and by biomass (90–95%) (Eastman, 2005) and evolved exceptional adaptations in response to an environment that is shaped by subzero water temperatures and the widespread presence of sea ice. A common characteristic of all notothenioids is the lack of a swim bladder. Therefore, most notothenioid species are negatively buoyant. To compensate for this morphological limitation, several notothenioid clades evolved adaptations to regain neutral buoyancy and are able to utilize (in addition to the ancestral benthic habitat) a set of different environments such as semipelagic, epibenthic, cryopelagic or pelagic habitats (Eastman, 2005). Morphological adaptations to enable the exploitation of these habitats include reduced mineralization of the skeleton and deposition of lipids in adipose cells (Balushkin, 2000; Eastman, 2000). These adaptations, probably together with diversification in body and head shape, enabled notothenioids to feed on a diverse diet. Stomach content analyses reveal a diet consisting of fish, krill and mysids for some species as well as polychaetes, ophiuroids and echinoderms for others (Rutschmann *et al*., 2011).

It is thought that the adaptive radiation of notothenioids followed ecological opportunity after the drop to subzero water temperatures around Antarctica that presumably led to the extinction of most of the previously existing ichthyofauna in the Late Oligocene or Early Miocene (Eastman, 1993; Near, 2004; Matschiner *et al*., 2011). Due to the emergence of antifreeze glycoproteins (AFGPs) in the ancestor of five predominantly Antarctic notothenioid families (the ‘Antarctic clade’) (Chen *et al*., 1997; Cheng *et al*., 2003), notothenioids of this particularly species-rich group were able to survive in subzero waters and could effectively exploit ecological niches that had become vacant. Thus, AFGPs may have acted as a key innovation, triggering the adaptive radiation of the notothenioid Antarctic clade (Matschiner *et al*., 2011), possibly facilitated by standing genetic variation (Brawand *et al*., 2014). However, a recent phylogenetic study (Near *et al*., 2012) found that major pulses of lineage diversification occurred substantially later than the evolution of AFGPs, which implies that other drivers were more important in driving diversification and acted at later stages. Thus, the timing and trigger of the notothenioid adaptive radiation remains a matter of debate.

Although the diversification of notothenioids has been the subject of a large number of recent investigations (Near & Cheng, 2008; Rutschmann *et al*., 2011; Near *et al*., 2012; Dettai *et al*., 2012), studies dealing with the morphology of notothenioid fishes using modern geometric morphometric approaches remain scarce. This type of analyses has previously been shown to be highly useful for the quantification of shape differences between specimens or species and to display these differences in a way that facilitates their interpretation in an evolutionary context. In a pioneering study, Klingenberg & Ekau (1996) investigated morphological changes associated with pelagic lifestyle in one of the notothenioid families. More recently, Wilson *et al*. (2013) assessed the shape of the operculum in a range of notothenioid species and correlated it with ecology and phylogenetic relationships. The study revealed a broad diversity of opercle morphologies with clear clustering according to phylogenetic groups as well as a correlation of opercle shape with the position along the benthic–pelagic axis. The authors used a broad taxon sampling including four of five families of the notothenioid Antarctic clade, but found no support for an early burst of opercle variation.

Here, we use a new time-calibrated phylogeny of 49 notothenioid species to test for patterns of taxonomic diversity as well as morphological and ecological disparity over time in the adaptive radiation of Antarctic notothenioids. Although our phylogeny includes less notothenioid taxa than previously published phylogenies (Near *et al*., 2012), it is based on an extensive comparison of models for Bayesian phylogenetic inference, including models with unlinked gene trees (i.e. the multispecies coalescent approach of *BEAST; Heled & Drummond, 2010), and may thus provide a more accurate picture of notothenioid diversification. We investigate disparity through time (DTT) in multiple ecologically important traits, including body shape, body size, buoyancy adaptations and habitat preferences as approximated by temperature range within species' geographic distributions. We find that notothenioids of the Antarctic clade are characterized both by elevated diversification rates and by an early burst in trophic morphology, thus supporting the adaptive nature of their radiation.

## Materials and methods

### Sample collection and DNA sequencing

We collected 703 individuals of 42 notothenioid species with bottom and pelagic trawls during two Antarctic expeditions with RV Polarstern in the austral seasons 2010/2011 and 2011/2012 (ANT-XXVII/3 and ANT-XXVIII/4). For 34 species, muscle tissue was extracted from two to three specimens and stored in 95% ethanol until DNA extraction. For *Dissostichus eleginoides*, a freshly caught specimen was obtained at a local fish market in Buenos Aires, Argentina, in November 2009, of which tissue was extracted and stored in the same way.

Genomic DNA was obtained from notothenioid muscle tissue by proteinase *K* digestion followed by sodium chloride extraction and ethanol precipitation. Up to two mitochondrial and four nuclear protein-coding markers (in genes mt-cyb, mt-nd4, enc1, myh6, PTCHD4 and tbr1b) were amplified and Sanger-sequenced on an ABI3130*xl* capillary sequencer (Applied Biosystems, Foster City, CA, USA) with conditions as described in Matschiner *et al*. (2011) and Rutschmann *et al*. (2011). See Table S1 for details including primer sequences and marker references. Sequence base calls were performed with CodonCode Aligner v.4.2.4 (CodonCode Corporation, Centerville, MA, USA) and verified by eye. For each species, we used only sequence data of the individual that provided the best sequencing results. All sequence accession numbers are given in Table S2. Our molecular data set for 35 notothenioid species was complemented with sequences obtained from GenBank and the Barcode of Life Data System (BOLD) (Ratnasingham & Hebert, 2007) to result in a total of four mitochondrial (mt-co1, mt-cyb, mt-nd2 and mt-nd4) and seven nuclear (enc1, myh6, PTCHD4, rps7, snx33, tbr1b, zic1) sequences for 49 notothenioid taxa, with only 19 of 539 (3.5%) sequences missing (see Table S2).

For all 42 species caught during Antarctic field expeditions, up to 61 individuals (see Table S3) were photographed for morphometric analyses using a Nikon D5000 digital camera (Nikon Corporation, Tokyo, Japan) and a tripod. Photographs were always taken of the left side of each specimen with fins spread out, lying on a flat surface while minimizing bending. The camera lens was aligned horizontally to the surface. Overall, the 49 included taxa represent all eight nominal families of notothenioids and 33 of 44 recognized notothenioid genera (Eastman & Eakin, 2000). Species in our data set cover the known notothenioid sizes range, depth and geographic distribution, trophic levels and a variety of different life styles. Our taxon set therefore provides a representative sample of the morphological and ecological diversity found in notothenioids.

### Species tree reconstruction

For each marker, sequences were aligned with MAFFT v.7.122b (Katoh & Toh, 2008) using the ‘—auto’ option. Alignments were visualized with Mesquite v.2.75 (Maddison & Maddison, 2009), trimmed to start and end with first and third codon positions, and protein translations of all sequences were checked for stop codons. Finally, we removed phylogenetically uninformative insertions as well as questionable alignment positions adjacent to insertions.

The marker set was partitioned using the programs Concaterpillar (Leigh *et al*., 2008) and PartitionFinder (Lanfear *et al*., 2012) as described in Text S1. Bayesian species tree reconstructions were performed with BEAST v.2.1 (Bouckaert *et al*., 2014) under a wide range of models, including the reversible-jump-based (RB) substitution model implemented in the RB add-on for BEAST 2 (Bouckaert *et al*., 2013; Drummond & Bouckaert, 2014). Gene trees of individual markers were assumed to be linked or unlinked (using the multispecies coalescent model of *BEAST; Heled & Drummond, 2010) in separate analyses. Clock models were time-calibrated using three secondary divergence age constraints, and support for each model combination was assessed *a posteriori* using the Akaike information criterion through Markov chain Monte Carlo (AICM) analysis (Raftery *et al*., 2007). Species reconstruction details are given in Text S2.

After discarding the first 10% of MCMC generations as burn-in, posterior tree samples of replicate analyses were combined and summarized in maximal clade credibility (MCC) trees with ‘common ancestor’ node heights (Heled & Bouckaert, 2013). All BEAST analyses were repeated with mitochondrial or nuclear markers separately. To account for phylogenetic uncertainty in analyses of diversification rate and DTT (see below), we produced a set of 1000 trees that was sampled at random from the posterior tree distribution obtained with the full data set, combining both mitochondrial and nuclear markers, and with the best supported model combination according to AICM. For analyses that required a manual step for each tree (i.e. summarizing the positions of rate shifts inferred with the MEDUSA method, see below), the set of 1000 posterior trees was subsampled to yield a second set of 100 trees. BEAST XML files for with all model specifications, as well as posterior sets of 100 and 1000 species trees are deposited in Dryad (doi:10.5061/dryad.5jt5j).

As a second tool for species tree inference, we applied maximum pseudolikelihood for estimating species trees (MP-EST; Liu *et al*., 2010). To use this method, we first produced gene trees for each marker with RAxML v.8.0.26 (Stamatakis, 2006), using partitioning schemes determined by PartitionFinder for the GTR+Gamma model of sequence substitution, which was also used in RAxML. For each marker, 100 bootstrap replicate trees (Felsenstein, 1985) were generated, and these were used to produce 100 species tree replicates with MP-EST. As MP-EST allows only a single outgroup, we removed sequences of *Cottoperca trigloides* from each alignment prior to the RAxML tree inference, leaving *Bovichtus diacanthus* as the only representative of the notothenioid family Bovichtidae. This family was previously shown to be the sister of all other notothenioids (Matschiner *et al*., 2011; Near *et al*., 2012) and was therefore used as outgroup for phylogenetic inference. The 100 bootstrap replicate species trees were summarized in a majority-rule consensus tree with a low majority requirement of 10% to obtain the bifurcating tree topology best supported by bootstrap values. To statistically compare this tree topology with posterior tree samples from our BEAST analyses, we reran the BEAST analysis based on the best-supported model according to AICM (which included unlinked gene trees, see Results), but this time constraining the BEAST species tree to match the tree topology of the MP-EST consensus tree.

### Diversification rate analyses

To test for diversification rate shifts during the notothenioid radiation, we trimmed all time-calibrated species trees so that nearly the entire extant diversity of the notothenioid suborder could be assigned to the remaining tips, as listed in Table S8. The resulting diversity trees were analysed with the MEDUSA method (Alfaro *et al*., 2009) implemented in the R package GEIGER (Harmon *et al*., 2008) to estimate background speciation and extinction rates, and to identify clades with potentially elevated or decreased diversification rates. We expected to observe a single main increase in diversification at or near the base of the AFGP-bearing Antarctic clade of notothenioids, which is usually considered as the ‘notothenioid radiation’ (Eastman, 2005; Matschiner *et al*., 2011; Near *et al*., 2012) as it encompasses nearly the entire notothenioid species richness (122 of 132 species from five of eight families), including the morphologically most specialized groups. In addition, the notothenioid family Artedidraconidae has previously been shown to be exceptionally species rich given its age (Near *et al*., 2012) and could support a second rate shift in our phylogeny. Diversification rate analyses were conducted with the diversity tree corresponding to the MCC tree resulting from the best-supported model combination, for the tree resulting from rerunning the same model in BEAST with the topological constraint of the MP-EST species tree and with 100 diversity trees based on the set of 100 trees sampled from the posterior distribution of the same BEAST analysis. For effective calculation, we allowed models to contain a maximum of 15 rate shifts, a number that we expected to be much larger than the actual number of shifts. Models assuming different numbers of rate shifts were compared on the basis of their Akaike information criterion corrected for sample size (AICc), and rate shifts were retained whenever they led to improved AICc scores.

Following Near *et al*. (2012; also see Dornburg *et al*., 2008), we further calculated per-stage floating Kendall–Moran estimates of notothenioid diversification rates. To allow direct comparison with the results of Near *et al*. (2012), we used the same geological time intervals for these analyses: Late Miocene (subdivided into Tortonian, 11.6–7.2 Ma, and Messinian, 7.2–5.3 Ma), Early Pliocene (Zanclean, 5.3–3.6 Ma), Late Pliocene (Piacenzian, 3.6–2.6 Ma) and Pleistocene (2.6–0 Ma). We did not repeat these analyses for the Early and Middle Miocene, as these intervals would have (partially) predated the diversification of Antarctic notothenioids according to our age estimates. Kendall–Moran rate estimates were calculated for the MCC tree, the tree based on the MP-EST topology, and the posterior sample of 1000 trees resulting from the best-supported model combination, and in each case using both the full tree including bovichtid, pseudaphritid and eleginopid outgroups, and a trimmed tree reduced to representatives of the Antarctic clade. To account for missing taxa in our notothenioid phylogeny, Kendell–Moran diversification rates of notothenioids were compared to rates calculated in the same way for simulated phylogenies that were constrained to be equally old and species rich as Notothenioidei, and were trimmed to the number of species included in our phylogenies. Phylogenetic simulations were performed using Yule and birth–death models of diversification, and tree trimming was performed according to random sampling and a new ‘semidiversified’ sampling scheme (see Texts S3 and S4).

### Geometric morphometric measurements of body shape

To test for differences in the overall body shape between notothenioid species, we performed geometric morphometric analyses on the basis of digital images. Body shape was quantified in a set of 703 specimens representing 42 ecologically diverse high Antarctic species from the five Antarctic notothenioid families (see Table S3) using 18 homologous landmarks (see Fig. S1).

Body shape variation was digitized using tpsDIG v.2.17 (Rohlf, 2013) and analysed with MorphoJ v.1.06a (Klingenberg, 2011). We performed a canonical variate (CV) analysis, a method that maximizes between-group variance in relation to within-group variance, with species as the grouping criterion to show shape changes associated with shape differences between species. Shape changes were visualized using an outline shape approach as implemented in MorphoJ. Species means for the first two CVs were illustrated in a phylomorphospace plot produced with the R package phytools (Revell, 2011) and used for analyses of DTT following Harmon *et al*. (2003) (see below).

### Habitat characterization

To approximate the geographic distribution of individual notothenioid species, georeferenced point occurrence data were downloaded from Fishbase (Froese & Pauly, 2013), which represent a compilation of entries made to the Global Biodiversity Information Facility (GBIF; http://www.gbif.org), the Ocean Biogeographic Information System (OBIS; http://www.iobis.org) and Fishbase itself. To reduce overrepresentation of heavily sampled locations (Ready *et al*., 2010), point occurrence data were summarized as presence or absence in a grid of 0.5^°^ latitude/longitude cell dimensions. With the exception of bovichtid outgroups (*n* = 5) and *Harpagifer antarcticus* (*n* = 8), each notothenioid species included in our data set was present in at least 24 (*Parachaenichthys georgianus*) and up to 292 (*Pleuragramma antarctica*) grid cells (mean = 79.5; Table S9).

For each of these grid cells, environmental parameters were obtained from the AquaMaps database (http://www.aquamaps.org; Kaschner *et al*., 2013), a database designed for the prediction of global distributions of marine species. These predictions are made on the basis of a characterization of the environmental preferences of each species, and the database authors selected bottom depth, water temperature, salinity, primary production and sea ice concentration as five parameters that were best suited to quantify these preferences for marine species (Kaschner *et al*., 2006). As our study aims to investigate ecological niche partitioning during the notothenioid radiation, its incentives differ from those of the AquaMaps database, that is the prediction of species distributions. However, both approaches require a detailed characterization of the ecological niche occupied by a species, and thus, we consider the same parameters that have shown useful for the purpose of the AquaMaps database (Ready *et al*., 2010) as suitable proxies to study partitioning of ecological niches in marine taxa. For grid cells occupied by notothenioid taxa, we found very little between-species variation in bottom depth, salinity and primary production and, unsurprisingly, a strong correlation between sea ice concentration and water temperature (Fig. S2). Therefore, we here use water temperature as the only environmental parameter to characterize notothenioid habitats. Temperature data stored in the AquaMaps database represent sea surface temperatures extracted from the Optimum Interpolation Sea Surface Temperature atlas (http://www.esrl.noaa.gov/psd/data/gridded/data.noaa.oisst.v2.html) and are averaged over the period 1982–1999 (Ready *et al*., 2010; Kesner-Reyes *et al*., 2012). Sea surface temperature may deviate from the actual temperature experienced by notothenioid species in benthic habitats; however, depth-specific temperature data are not available from the AquaMaps database. Thus, we here use sea surface temperature for all notothenioid species, assuming that differences between this measure and the actual temperature in notothenioid habitats are minor compared to those observed between different species. As a result of this approximation, patterns inferred for the evolution of habitat preferences among notothenioids may need to be interpreted with caution.

In addition to sea surface temperature, we use species-specific buoyancy measures (taken from Near *et al*., 2012) as a second proxy to characterize notothenioid habitats. As notothenioid fishes possess no swim bladder, their position in the water column is directly influenced by adaptations to regain neutral buoyancy such as reduced mineralization of the skeleton and scales or the accumulation of lipid deposits (Eastman, 2000). Thus, buoyancy measures are informative regarding the depth distribution and the lifestyle of notothenioid species.

### Disparity through time

Analyses of morphological DTT indicate how the trait space occupied by a clade became partitioned during the diversification of the clade (Foote, 1999; Harmon *et al*., 2003). In this type of analyses, the observed DTT trajectory is usually compared to that expected according to pure Brownian motion (BM) (Harmon *et al*., 2003, 2008), and the difference between these is quantified by the morphological disparity index (MDI). Highly negative MDI values are commonly interpreted as evidence for an early burst in trait evolution in the investigated clade, supporting the adaptive character of the diversification process in this clade (Harmon *et al*., 2010; Slater *et al*., 2010; Slater & Pennell, 2014). However, the signature of an early burst, as measured by MDI, might be blurred by other processes that are characteristic of adaptive radiation. If the radiation proceeds in stages, as has been shown for several groups (Richman, 1996; Streelman & Danley, 2003; Gavrilets & Losos, 2009), early bursts would likely only occur along the first axis of diversification. Furthermore, the trait space for many characters may be bounded by hard or soft constraints, so that the evolution of these characters may be poorly approximated by a BM model. Among the parameters here investigated for notothenioid species, hard trait space bounds are obviously present for the temperature of sea water, which usually freezes at −1.86 °C (Eastman, 1993), and similar limits can be assumed for buoyancy values of fishes without swim bladders. In addition, soft bounds have been shown to limit the evolution of body size and shape in a wide range of vertebrate species (Harmon *et al*., 2010; Gherardi *et al*., 2013) and may therefore also be present in notothenioids.

To assess the impact of stagewise adaptive radiation and bounded trait spaces on DTT trajectories and their associated MDI values, we conducted simulations of trait evolution according to these more complex models on a large number of simulated phylogenetic trees. Specifically, we generated 2000 replicate trees using a continuous-time pure birth model with speciation rate *λ* drawn at random from a uniform distribution between 0.1 and 0.4, an extant species richness of exactly 100 taxa and a most recent common ancestor age of 15 million years (trees that did not fulfil these criteria were discarded). We used the Ornstein–Uhlenbeck (OU) model (Hansen, 1997; Butler & King, 2004) for trait evolution, applying a range of values for the constraint parameter *α* between *α *= 0 (in this case, the OU model is identical to BM) and *α *= 0.3. Positive values of *α* influence the long-term behaviour of traits and can be interpreted as soft trait space bounds or selection towards an optimum (in our simulations, optimum and starting value were always chosen to be both 0). Different stages of adaptive radiation were simulated by 10-fold elevated rates of trait evolution in the first 5 million years of the radiation (15–10 Ma), the second interval of 5 million years (10–5 Ma) and the period between 5 Ma and the present. The first of these three scenarios is similar to the early burst model of Harmon *et al*. (2010) in having an elevated initial rate of trait evolution, but contrary to the early burst model, the rate does not decline continuously, but with a single, abrupt decrease at 10 Ma. Finally, all simulated data sets were subjected to DTT analyses, and MDI values were calculated on the basis of 100 BM simulations, both using the function dtt() implemented in GEIGER.

We compared DTT plots of simulated trait evolution with those of observed traits characterizing the morphometry and habitat of notothenioid fishes. Here, morphometry of individual species was described by the mean values of the first two canonical variates of body shape variation and by mean body size measured as terminal length. The interspecific variation in habitat use was described by buoyancy measures and by mean sea surface temperature of geographic grid cells in which a species is known to occur (see above). Species means of all five traits are compiled in Table S3. For each trait, we first determined whether a BM model or an OU model of trait evolution provided a better fit to the observed values. Parameters of the two models were optimized for each trait and for each of the 1000 trees drawn from the BEAST posterior tree distribution using the maximum likelihood function fitContinuous() of GEIGER. Per trait and tree, model fit was compared on the basis of AICc scores. The observed DTT curves of the five notothenioid traits were contrasted with DTTs simulated with the best-fitting model of trait evolution, for the same set of trees. Finally, we compared DTT distributions resulting from the extensive set of simulations described above with those based on trait variation observed in notothenioids, both qualitatively by visual inspection and quantitatively by means of the associated MDI values. Note that for comparability between analyses, all MDI values were calculated as the area between a DTT and the median average subclade disparity in 100 simulations of BM trait evolution in the same tree as the DTT.

## Results

### Species tree reconstruction

Applying gene tree concordance tests with the software Concaterpillar, we found no significant discordance among nuclear markers. However, different evolutionary histories were detected for the concatenated nuclear alignment and the combined mitochondrial marker set (likelihood ratio test based on nonparametric bootstrapping; *P* < 0.001). Maximum likelihood trees resulting from these two alignments differ strongly in their topologies (Fig. S3), and one of the most obvious differences concerns the placement of *Gobionotothen*, which is the first lineage to diverge among Antarctic notothenioids in the mitochondrial tree, but appears nested within this clade based on nuclear data.

Judging from a comparison of parameter traces in Tracer v.1.5 (Rambaut & Drummond, 2007), replicate BEAST runs always converged to the same solution. After discarding the burn-in, ESS values of likelihood traces (the only traces needed for model comparison by AICM), combined for all replicates, were > 700 for all analyses, and ESS values of model parameters were almost always > 200. According to AICM values, the most parameter-rich model combination, the RB+Gamma substitution model with estimated frequencies and a UCLN clock, outperformed all other models in all analyses, regardless of whether gene trees were linked or unlinked, and with all data sets (combined, mitochondrial, and nuclear). Linking of gene trees led to better AICM values only for the nuclear data set, suggesting that with this data set, the large increase in parameter number outweighs the obtained improvements in the likelihood when gene trees topologies are unlinked. Akaike weights computed from AICM values strongly favoured unlinked gene trees for the combined (Akaike weight = 1.0) and mitochondrial data sets (Akaike weight = 0.997), and supported linking of gene trees for the nuclear data set (Akaike weight = 1.0) (Tables S4–S7).

Both the topology and branch lengths of resulting MCC trees were largely dependent on the assumed models. As a relatively high number of substitutions in nuclear markers separates *P. antarctica* from other Antarctic notothenioids, all MCC trees based on the nuclear marker set and a strict molecular clock inferred *Pleuragramma* to be the earliest diverging lineage among Antarctic notothenioids, with mean separation times of 19.1–14.9 Ma. The same was true for analyses of the combined marker set, but only when gene trees were unlinked (18.7–17.6 Ma). In contrast, almost all MCC trees based on either the combined or nuclear marker set and the UCLN molecular clock identified the earliest divergence among Antarctic notothenioids between two clades, where the first of these clades contains the nototheniid subfamily Trematominae (Lautrédou *et al*., 2012) and the second clade groups the four more derived families Artedidraconidae, Harpagiferidae, Bathydraconidae and Channichthyidae with other nototheniid lineages. Here, the exception was the MCC tree based on the nuclear marker set, the RB+Gamma substitution model, estimated base frequencies and a UCLN clock, in which *Aethotaxis mitopteryx* diverges first from other Antarctic notothenioids (11.2 Ma), followed by *P. antarctica* (10.4 Ma).

**Figure 1:**
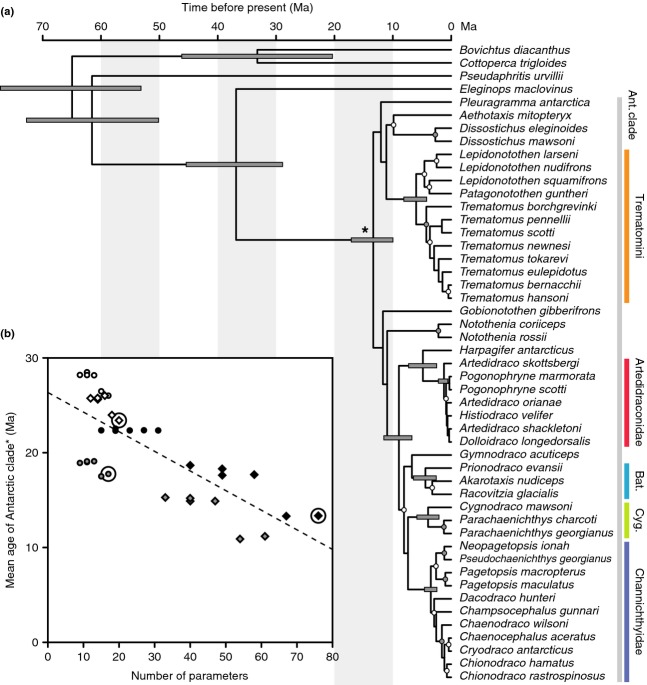
Time-calibrated species tree of Notothenioidei. (a) The maximal clade credibility (MCC) tree for the BEAST analysis of the combined marker set and the best-supported model combination. Node bars are only shown for clades supported by Bayesian posterior probability (BPP) 1.0 and indicate the divergence date 95% highest posterior density. Gray circles mark nodes supported with BPP > 0.9 and white circles indicate BPP > 0.5. Support values and age estimates for all nodes are listed in Table S10. Vertical colour bars at right indicate monophyletic clades. Bat.: Bathydraconinae; Cyg.: Cygnodraconinae. The vertical gray bar spans the Antarctic clade. A lineage-through-time plot for this MCC tree is shown in Fig. S6. (b) The mean age estimate for the diversification of Antarctic notothenioids (the node marked with *) plotted against the number of parameters used in the respective BEAST analysis. Analyses using unlinked gene trees are represented by diamonds, and those with linked gene trees are marked with circles. Fill colours of circles and diamonds indicate the marker set used for the analysis (white: mitochondrial, gray: nuclear, black: combined). The best-supported model combination for each data set is encircled.

Inferred ages for the onset of the divergence of Antarctic notothenioids (the node marked with * in Fig.1a) varied strongly between the individual analyses with mean estimates between 10.9 Ma (95% highest posterior density, HPD: 14.6–7.9 Ma) and 28.5 Ma (95% HPD: 33.6–23.5 Ma). As expected, ages inferred with unlinked gene trees were always younger than those based on analyses with linked gene trees. We generally observed lower mean age estimates for more parameter-rich models and found a significant negative correlation between the number of parameters present in the model and the mean age inferred for the first divergence event of Antarctic notothenioids (*b* = −0.207 myr/parameter; *t*_34_ = −6.918, *P* < 0.001, *r*^2^ = 0.57) (Fig.1b).

According to AICM, the best model for analyses of the combined marker set applies the RB+Gamma substitution model with estimated base frequencies and a UCLN molecular clock to unlinked gene trees. This is the most parameter-rich of all used models (76 parameters) and, consequently, required one of the longest MCMC lengths to obtain sufficiently high ESS values (nine replicates with 2 billion MCMC steps each, the first 50% of each replicate were discarded as burn-in). The resulting age estimate for the initial divergence of Antarctic notothenioids is lower than in most other analyses, with a mean of 13.4 Ma and a 95% HPD interval between 17.1 and 10.0 Ma. The corresponding tree topology strongly supports the monophyly of Antarctic notothenioids (Bayesian posterior probability, BPP 1.0), but within this clade, only few groups receive equally strong support. These include Trematominae (Lautrédou *et al*., 2012), the families Artedidraconidae and Channichthyidae, and the two bathydraconid subfamilies Bathydraconinae and Cygnodraconinae (Derome *et al*., 2002; see also Near *et al*., 2012). In agreement with previous studies (Matschiner *et al*., 2011; Dettai *et al*., 2012; Near *et al*., 2012), the same tree further supports a clade combining the four most derived Antarctic families Artedidraconidae, Harpagiferidae, Bathydraconidae and Channichthyidae, as well as a sister-group relationship of Harpagiferidae and Artedidraconidae (both groupings receive BPP 1.0). In contrast to the phylogeny of Near *et al*. (2012), we find no support for Pleuragrammatinae (Balushkin, 2000) as a nototheniid subfamily combining the pelagic genera *Pleuragramma*, *Aethotaxis* and *Dissostichus* (as well as *Gvozdarus*, which is missing from our data set). The monophyly of the family Harpagiferidae and the bathydraconid subfamily Gymnodraconinae (Derome *et al*., 2002) could not be tested as our data set included only a single representative of both groups.

Despite relatively low bootstrap support, the bootstrap consensus species tree topology obtained with MP-EST agrees well with the MCC tree resulting from the BEAST analysis with the best-supported model combination (Fig. S4). The most noticeable differences include the placement of *Gobionotothen gibberifrons*, which appears as the sister of all other Antarctic notothenioids in the MP-EST species tree, and the placement of Bathydraconinae instead of Cygnodraconinae as the sister group of Channichthyidae. However, both rearrangements receive low support in the MP-EST species tree [bootstrap support (BS) 49 and 54, respectively]. Rerunning the best-supported model combination in BEAST, using the MP-EST species tree topology as a topological constraint, results in very similar age estimates compared to the MCC tree from the unconstrained BEAST run with the same model combination (Fig. S5a). The posterior probability distribution of the topologically constrained analysis was nearly identical to that of the unconstrained runs (Fig. S5b), suggesting that the MP-EST species tree topology was also within the posterior tree distribution of the topologically unconstrained BEAST analysis.

### Diversification rate analyses

To identify diversification rate shifts during the evolution of notothenioid fishes, we applied MEDUSA to a diversity tree resulting from the MCC tree based on the BEAST analysis with the best-supported model combination (Fig.1a), to the tree resulting from a reanalysis of the same model combination, but using the MP-EST species tree as a topological constraint (Fig. S5), and to a set of 100 diversity trees that account for the uncertainty in the phylogenetic estimate resulting from the topologically unconstraint BEAST analysis. In the diversity tree based on the MCC tree, MEDUSA identified a single rate shift at the base of the divergence of all Antarctic notothenioids (the node marked with * in Fig.1a) that led to an improvement in AICc score of 11.7 units. Maximum likelihood estimates for net diversification (*r*) and turnover rates (*ε*) were *r* = 0.029 and *ε *= 0.030 per myr for non-Antarctic notothenioids, and *r* = 0.106 and *ε* = 0.887 per myr for Antarctic notothenioids subsequent to the inferred shift. Use of the MP-EST species tree as a topological constraint also resulted in a single rate shift (from the background rates of *r* = 0.009 and *ε *= −0.882 to *r* = 0.251 and *ε *= 0.614); however, in this case, the rate shift excludes the genus *Gobionotothen*, which appears as the sister of all other Antarctic notothenioids in the MP-EST topology. In the set of 100 diversity trees, MEDUSA identified a single rate shift in 61 of these trees, two rate shifts in 33 trees and three rate shifts in six trees. One of the shifts always preceded or coincided with the separation of Trematominae from the four families Artedidraconidae, Harpagiferidae, Bathydraconidae and Channichthyidae, and led to elevated diversification rates of *r* = 0.215 ± 0.091, compared to background rates of *r *=* *0.020 ± 0.008. However, only in 37 of the 100 trees were all Antarctic notothenioids affected by this shift. In the remaining 63 trees, one or several of the nototheniid lineages *Aethotaxis* (in 51 trees), *Dissostichus* (48 trees), *Pleuragramma* (34 trees), *Gobionotothen* (13 trees)*, Notothenia* and *Paranotothenia* (both in three trees) diverged before the separation of Trematominae and the four more derived families, and were not included in the same diversification regime. Thus, uncertainty remains whether these lineages should be considered part of the same radiation as Trematominae, Artedidraconidae, Harpagiferidae, Bathydraconidae and Channichthyidae.

As expected, Kendall–Moran estimates of per interval diversification rates were generally lower for trees of all notothenioids included in our taxon set, compared to trees reduced to representatives of the Antarctic clade (Fig. S7). In all time intervals of the Lower Miocene and the Pliocene, trees of the Antarctic clade had comparable diversification rates to simulated phylogenies of the same age and species richness. The exception to this is the Pleistocene, where rate estimates for the Antarctic clade appear high compared to those in simulated phylogenies: when simulations were based on a strict Yule model, the rate estimates for the MCC tree and the tree based on the MP-EST topology, as well as the mean rate in a sample of 1000 trees, were higher than the 99.9% quantile of rates found in simulated phylogenies after application of the semidiversified sampling scheme (Fig. S7e). Compared to phylogenies simulated with a birth–death model, however, these rates appear less exceptional, and only the mean rate in the sample of 1000 trees and the rate of the tree based on the MP-EST topology, but no longer the rate of the MCC tree, were higher than the 95% quantile of rates in simulated phylogenies. This pattern is strikingly different when trees of all notothenioids in our taxon set are compared to simulated trees of the same age and species diversity as Notothenioidei, after trimming these simulated trees to match the number of taxa in our empirical phylogeny, again with a random or semidiversified sampling scheme. In this case, diversification rate estimates in observed trees are higher than the 95% quantiles of rates in simulated trees in almost all tested time intervals, regardless of whether the Yule or birth–death model is used for phylogenetic simulations.

### Geometric morphometrics

**Figure 2:**
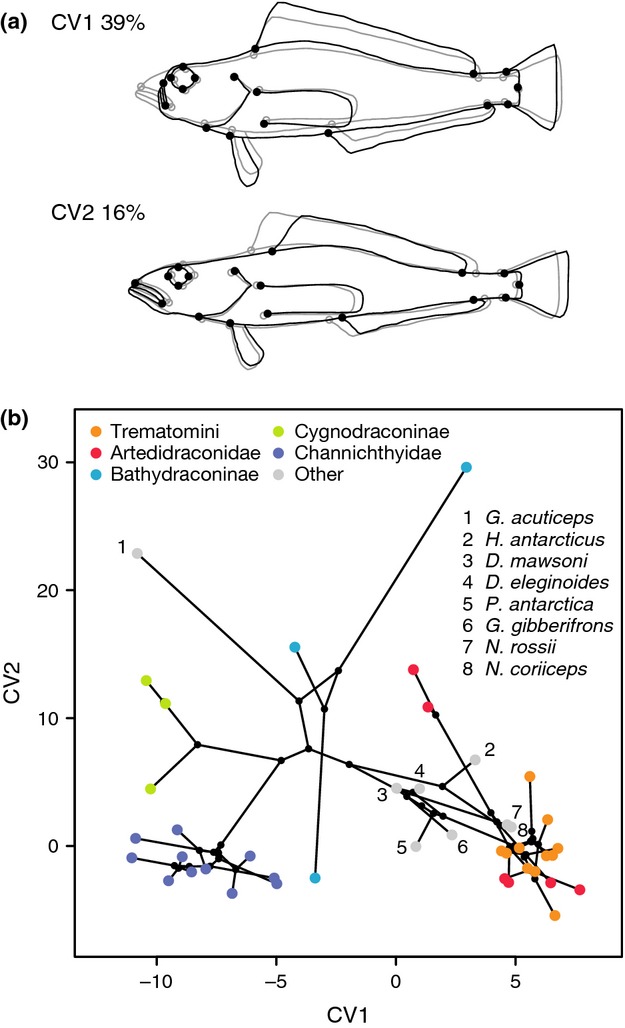
Body shape variation in notothenioid species. (a) Shape changes along the first two canonical variates. (b) Phylomorphospace plot of the first two canonical variates of body shape variation. Coloured dots show mean values of notothenioid species, whereas dot colour indicates clade membership. Colour code as in Fig.1a. Species not assigned to clades are represented by gray dots, with labels indicating species names. Black lines show phylogenetic relationships, and black dots represent hypothesized ancestral trait values.

The first two CVs (Fig.2a) account for ∼55% of the total variance in the body shape data set. CV1 (∼39% of variance) illustrates a shape change towards a shorter, more compressed snout with the mouth facing upward and a deeper body, mainly concerning the abdomen but also the tail and caudal peduncle. The onset of the anal fin is slightly shifted anterior, as is the pelvic fin, whereas the dorsal fin is shifted posterior. Shape change in CV2 (∼16% of variance) is associated with a deeper snout, the eye shifted anterior and both shorter dorsal and anal fins, whereas the caudal peduncle is slightly elongated.

A phylomorphospace plot (Fig.2b) including the first two CVs shows a clustering according to taxonomy for some families, whereas others show a more diverse body shape distribution. CV1 mainly discriminates between Channichthyidae and Cygnodraconinae characterized by a long, pikelike snout (negative CV1 scores) on one side and an overlapping cluster consisting of Nototheniidae, Artedidraconidae and Trematomini characterized by a shorter, more robust head (positive CV1 scores) on the other side. CV2, mainly associated with alterations of the unpaired fins, shows great variation in Bathydraconidae and Artedidraconidae, probably because some family members are characterized by strong reductions or even the complete loss of the first dorsal fin. Other families, such as Channichthyidae, show remarkably constant CV2 values.

### Habitat characterization

**Figure 3:**
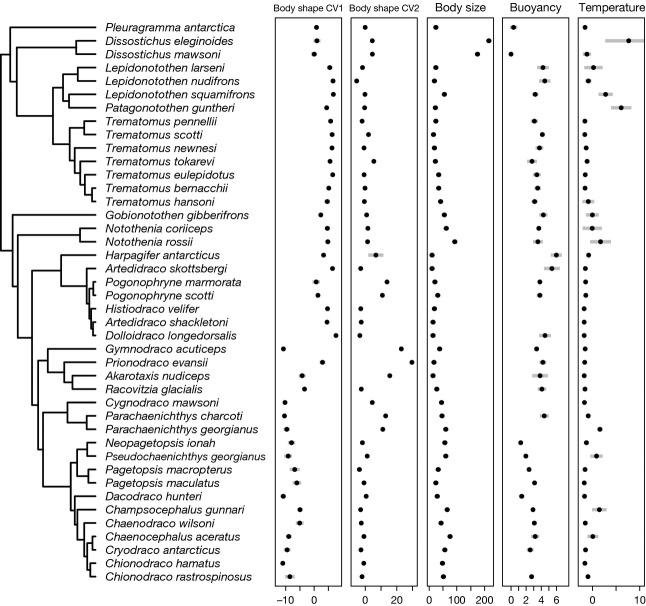
Variation in morphological and habitat characteristics among notothenioid species. For each species, black dots indicate means of observed trait values, and gray bars represent standard variation. Body size is measured in cm, buoyancy in per cent, and temperature in °C. Buoyancy values are taken from Near *et al*. (2012). The phylogenetic tree is identical to the one shown in Fig.1a, excluding species with missing trait data.

Sea surface temperature data were extracted from the AquaMaps database for geographical grid cells, in which notothenioid species are known to occur. As expected, sea surface temperature was found to correlate with latitude (*b* = −0.40 °C/degree south; *t*_966_ = −56.38, *P* < 0.001, *r*^2^ = 0.77), with a minimum temperature of −1.79 °C found in a total of 126 grid cells between 67.25˚S and −78.25˚S, in which 28 of the 42 notothenioid species in our trait data set are known to occur. These include *P. antarctica*, *Dissostichus mawsoni*, *Notothenia coriiceps*, seven of eight included members of the genus *Trematomus*, all seven included members of Artedidraconidae, the three included members of Bathydraconinae, *Gymnodraco acuticeps*, *Cygnodraco mawsoni* and seven of 11 included members of Channichthyidae. The maximum temperature of 19.25 °C was found at 34.75˚S, 51.75˚W, off the Uruguayan coast, which is the northern range limit of *D. eleginoides*. Mean temperatures of grid cells occupied by members of selected notothenioid clades were between −1.62 °C (Bathydraconinae) and −0.51 °C (Trematominae), and temperature ranges of clades varied greatly (Trematominae mean: −0.51 °C, range: −1.79 to 9.11 °C; Artedidraconidae mean: −1.55 °C, range: −1.79 to 0 °C; Bathydraconinae mean: −1.62 °C, range: −1.79 to 0 °C; Cygnodraconinae mean: −0.70 °C, range: −1.79 to 3.23 °C; Channichthyidae mean: −0.89 °C, range: −1.79–3.99 °C). Water temperatures of individual species' ranges are shown in Fig.3.

### Disparity through time

**Figure 4:**
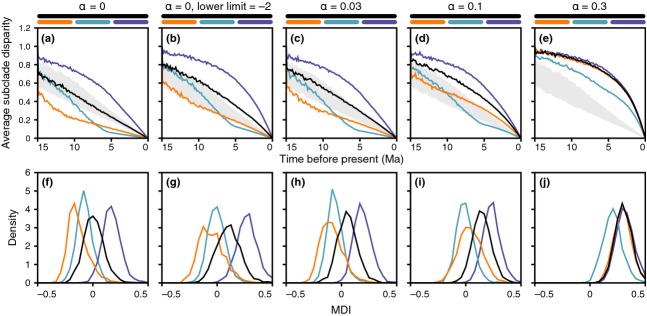
Disparity through time and associated morphological disparity index (MDI) values for simulated trait evolution. (a–e) Average subclade disparity over time in simulated phylogenies with an age of 15 myr. The horizontal axis represents time. The black line represents the mean value for 2000 replicates of simulated diversification and trait evolution when the rate of trait evolution is homogeneous. Orange, turquoise and blue lines indicate mean values when the rate of trait evolution is 10-fold elevated between 15 and 10 Ma, between 10 and 5 Ma, or between 5 Ma and the present. (f–j) Densities of MDI values in 2000 trait evolution replicates, in the same sequence as (a–e).

Analyses of DTT were conducted for a wide range of trait evolution simulations and compared to DTTs based on the observed characteristics for the morphology and habitat of notothenioid species. Simulations were performed with either homogeneous or periodically elevated rates of trait evolution that followed an Ornstein–Uhlenbeck (Hansen, 1997) model with a constraint parameter α between α = 0 (in this case, the Ornstein–Uhlenbeck model reduces to BM) and α = 0.3. Traditionally, DTT trajectories are compared to those obtained under a BM null model, and exceptionally low DTT trajectories are taken as evidence for early bursts of trait evolution. Plots in Fig.4 show mean DTT trajectories (in Fig.4a–e) and associated MDI values (Fig.4f–j) when traits evolved with a homogeneous rate or with a rate that is 10-fold higher in the beginning of a clade's diversification, during an intermediate interval, or near the present. As expected, average subclade disparities and the associated MDI values are generally lower when rates are initially elevated, compared to when rates remain constant throughout the clade's history, or are elevated near the present. However, the addition of hard or soft boundaries to trait evolution always leads to a shift to more positive average subclade disparities and MDI values, so that, depending on the position and strength of these boundaries, the signature of initially elevated rates becomes less obvious (Fig.4b,c) or disappears altogether (Fig.4d,e). As a result, early bursts may not be detectable when trait space is constrained by hard or soft boundaries. Thus, low observed average subclade disparities and MDI values are likely to indicate elevated rates early in a clade's history, whereas high disparities and MDI values can result from either elevated rates near the present or hard or soft boundaries to trait evolution.

**Figure 5:**
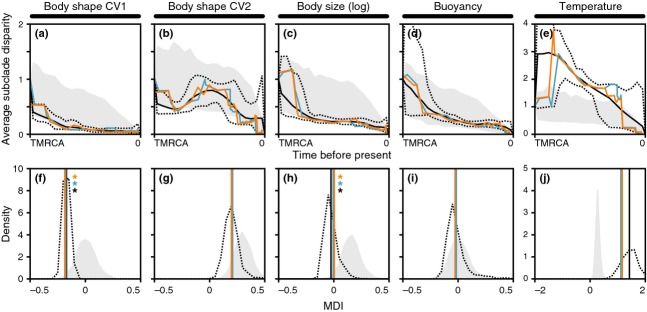
Disparity through time and associated morphological disparity index (MDI) values for observed notothenioid traits. (a–e) Average subclade disparity over time. The horizontal axis represents time between the time of the recent common ancestor (TMRCA) and the present. Solid and dotted black lines indicate the mean and the 5% and 95% quantiles for average subclade disparity found in a posterior tree sample of 1000 trees. The orange and turquoise lines mark average subclade disparity in the maximal clade credibility (MCC) tree and in the tree based on the MP-EST species tree topology, respectively. The gray area represents the 5% and 95% quantiles for average subclade disparity according to the fitted model of trait variation (see Table1). (f–j) Solid lines represent MDI values calculated over the first 90% of the chronogram to account for tip overdispersion (Harmon *et al*., 2003), with colour codes as in (a). The black dotted line shows the density of MDI values in the sample of 1000 trees. The gray shape shows the density of MDI values in 2000 trees simulated with the fitted model of trait evolution (BM for body shape CV1 and buoyancy, OU with *α *= 0.30, 0.11 and 0.25 for body shape CV2, log body size and temperature; see Table1). Asterisks in (f) and (h) indicate that the tree sample mean MDI, the MDI of the MCC tree and the MDI of the tree based on the MP-EST topology are lower than the 5% quantile of MDI values found with the fitted model. Note the different scales for (e) and (j).

Maximum likelihood model fitting showed that the OU model provided a better fit than BM to trait evolution for body shape CV2, for log body size and for the sea surface temperature of notothenioid habitats (Table1), with ML estimates of the *α* parameter between 0.11 (body size) and 0.30 (CV2). DTT curves and MDI densities for notothenioid body shape and size, as well as buoyancy and the sea surface temperature of notothenioid habitats, are shown in Fig.5. Regardless of clade age, average subclade disparities of CV1 are low compared to those simulated under BM (Fig.5a,f). Asterisks in Fig.5f indicate that for CV1, the MDI of the MCC tree (−0.188), the MDI of the MP-EST species tree after branch length optimization with BEAST (−0.177) and the mean MDI in a sample of 1000 trees (−0.169) are lower than the 5% quantile (−0.167) of MDI values under a BM expectation, thus suggesting that rates of evolution were high during the early evolution of Antarctic notothenioids. In contrast, MDI values for CV2 (MCC tree: 0.202, MP-EST species tree: 0.209, mean of tree sample: 0.223) are higher than the 95% quantile under BM (0.198), but agree well with an OU model of trait evolution (with *α *= 0.30; see Table1) (Fig.5b,g). Both the DTTs of log body size (Fig.5c,h) and buoyancy (Fig.5d,i) appear consistent with expectations from a BM null model; however, average subclade disparities and MDI values of log body size (MCC tree: 0.005, MP-EST species tree: 0.006, mean of tree sample: −0.026) are outside of expectations based on the fitted OU model (with *α *= 0.11) for this trait (5% quantile: 0.032), suggesting that despite the improved AICc score of the OU model, BM describes notothenioid body size evolution sufficiently well. Remarkably high average subclade disparities and MDI values were observed for the sea surface temperature of notothenioid habitats (Fig.5e,j; note the different scale). MDI values for this character (MCC tree: 1.169, MP-EST species tree: 1.114, mean of tree sample: 1.456) were higher than the highest recorded values in 2000 replicate simulations of lineage diversification and trait evolution, regardless of whether these were based on BM (maximum MDI: 0.538) or the fitted OU model (with *α *= 0.25; maximum MDI: 0.952). This pattern seems to be mostly driven by the comparatively late separation (mean age estimate: 2.72 Ma, 95% HPD: 5.47–0 Ma) of *D. mawsoni*, one of the more cold-adapted species (mean temperature of grid cells: −1.15 °C), and *D. eleginoides*, which seems to lack functional AFGP sequences (Cheng & Detrich, 2007) and has the most temperate distribution of all species included in our comparison (mean temperature of grid cells: 7.64 °C). Exclusion of *D. eleginoides* from DTT analyses leads to lower MDI values (MCC tree: 0.421, MP-EST species tree: 0.467, mean of tree sample: 0.408), which are still higher than the 99.5% quantile of MDI values under BM (0.338), but agree with the expectations of the fitted OU model (95% quantile: 0.509).

**Table 1:** Maximum likelihood fitting of Brownian motion (BM) and Ornstein–Uhlenbeck (OU) models to trait evolution of body shape, size, buoyancy and habitat temperature

Trait	Model	*σ*^2^	*α*	LnL	AICc	#Trees
Body shape CV1	BM	3.47		−112.67	229.65	627
	OU	4.11	0.04	−111.95	230.53	373
Body shape CV2	BM	18.15		−147.53	299.37	40
	OU	39.05	0.30	−140.80	288.24	960
Body size (log)	BM	0.08		−34.02	72.34	335
	OU	0.12	0.11	−31.90	70.43	665
Buoyancy	BM	0.23		−48.94	102.26	723
	OU	0.27	0.03	−48.70	104.17	277
Temperature	BM	1.18		−89.47	183.25	120
	OU	2.37	0.25	−84.75	176.12	880

Parameter values were optimized for the set of 1000 trees drawn from the posterior distribution of the BEAST run with the combined data set and the model combination best supported by AICM. To reduce the impact of outlier estimates, median values are given for *σ*^2^, *α*, the log likelihood and Akaike information criterion scores, corrected for small sample sizes (AICc). The last column specifies the number of trees for which a model had a lower AICc score than the competing model.

## Discussion

It has previously been shown that Antarctic notothenioids fulfil multiple criteria of adaptive radiation, including common ancestry, rapid diversification, phenotype–environment correlation, trait utility and convergent evolution (Schluter, 2000; Eastman, 2005; Cheng & Detrich, 2007; Bilyk & DeVries, 2010; Rutschmann *et al*., 2011). However, whether or not other predictions of adaptive radiation theory, such as early bursts in diversity and disparity (Gavrilets & Losos, 2009), are realized in Antarctic notothenioids remains a matter of debate. Here, we thus tested for temporally declining rates in the evolution of notothenioid diversity and disparity on the basis of a novel time-calibrated phylogeny of notothenioid fishes.

### The age of the notothenioid radiation

As it has often been hypothesized that the notothenioid radiation in the freezing waters of Antarctica is linked to the evolution of AFGPs (Eastman, 1993), numerous previous studies have attempted to time-calibrate the origin of this radiation to test for possible correlations with cooling events recorded from geological data. Based on fossil occurrences in non-notothenioid outgroup lineages, Matschiner *et al*. (2011) estimated the radiation onset near the Oligocene–Miocene boundary (mean age estimate 23.9 Ma), coincident with the Mi-1 cold event (Naish *et al*., 2001). The age estimates obtained by Matschiner *et al*. (2011) for the divergence of Bovichtidae, Pseudaphritidae, Eleginopsidae and the Antarctic clade were reused subsequently to time-calibrate the phylogenies of Rutschmann *et al*. (2011) and Near *et al*. (2012), which both used more extensive taxon coverage within Notothenioidei, but did not include non-notothenioid outgroups. As the latter two studies used age constraints on the age of the Antarctic clade, they arrived at mean age estimates very close to that of the applied constraint (24.2 and 22.4 Ma).

The phylogenetic hypotheses presented here (Fig.1a) are based on inference methods that differ in various aspects from those applied in previous studies of the notothenioid diversification. We produced sets of time-calibrated phylogenies for three different data sets and twelve different model combinations, including – for the first time – the application of the multispecies coalescent model of *BEAST (Heled & Drummond, 2010) to a phylogeny of Notothenioidei. We find that age estimates for the adaptive radiation are strongly dependent on the applied data set and model combination and that for two of three data sets, the most parameter-rich models, which have the drawback of substantially longer convergence times and generally lower node support, provide the best fit according to AICM values (Tables S4–S7). Furthermore, we find over all data sets and model combinations a significant negative correlation between age estimates and model complexity measured in numbers of parameters (Fig.1b).

Assuming that the BEAST analysis based on the combined marker set and the best-supported model combination (Fig.1a) provides a realistic time line of notothenioid diversification, the onset of the notothenioid adaptive radiation occurred around 13.4 Ma (95% HPD: 17.1–10.0 Ma), which is substantially younger than a previous estimate by Matschiner *et al*. (2011). As our model comparison shows (Fig1b), this difference can largely be attributed to different gene tree models, as Matschiner *et al*. (2011) used sequence concatenation and linked gene trees, whereas these remained unlinked in the best-supported model combination of the present study. Nevertheless, the contrasting time estimates of different studies highlight the importance of extensive model testing, including highly parameter-rich model combinations that can account for incomplete lineage sorting (Heled & Drummond, 2010).

If the present age estimates for the notothenioid radiation should be correct, its onset might have coincided with the Middle Miocene climatic transition (MMCT; 14.1–13.9 Ma), during which Southern Ocean sea surface temperatures declined by 6–7 °C (Shevenell *et al*., 2004) and a full polar climate became established in Antarctica (Lewis *et al*., 2008). This would support previous speculations that AFGPs evolved between 15 and 10 Ma (Eastman, 1993; Bargelloni *et al*., 1994) and that the notothenioid radiation was triggered by ecological opportunity following the extinction of less cold-adapted teleost fishes and the availability of new habitats associated with sea ice (Eastman, 1993; Matschiner *et al*., 2011).

### Diversification rates over time in the notothenioid radiation

In agreement with earlier studies (Near *et al*., 2012), diversification rate analyses with MEDUSA strongly supported a primary rate increase at or near the base of the Antarctic clade. Regardless of whether or not individual lineages, such as *Aethotaxis* and *Pleuragramma* diverged before this event, the observed rate shift supports the view that Antarctic notothenioids represent an adaptive radiation. By comparing time-interval-specific Kendall–Moran diversification rate estimates for an inferred notothenioid phylogeny with those found in simulated phylogenies of the same age and species richness, Near *et al*. (2012) found exceptionally high notothenioid diversification rates only at later stages of the radiation. Based on these results, the authors argued that the bulk of the notothenioid diversity originated long after initial divergences within the Antarctic clade. This disagrees with the notion of Antarctic notothenioids as a case of adaptive radiation, in which diversification is driven by available ecological niche space and speciation rates decrease as more and more niches are occupied. Using the same approach as Near *et al*. (2012), we obtained similar results when phylogenies of notothenioids, including Bovichtidae, Pseudaphritidae and Eleginopsidae, were compared to simulated phylogenies conditioned on the age and extant species richness of Notothenioidei, and resampled to match the number of taxa present in our taxon set. However, at least one positive rate shift event is consistently identified during the notothenioid diversification, at the base of the Antarctic clade (Near *et al*., 2012; this study). Thus, a comparison with phylogenies simulated under homogeneous rate models is necessarily biased so that empirical rates in early time intervals appear low compared to rates in simulated trees and empirical rates subsequent to the shift appear high in comparison. Consequently, it may be more appropriate to account for the observed rate shift and directly compare empirical phylogenies that are trimmed to include only taxa descending from the rate shift with phylogenies that are simulated correspondingly. When notothenioid phylogenies are reduced to include only the Antarctic clade, and simulated phylogenies are conditioned on the age and species richness of this clade (and subsequently resampled to match the number of taxa in our data set), a time interval-specific comparison yields no evidence of diversification bursts during the late Miocene (11.6–5.3 Ma) or Pliocene (5.3–2.6 Ma) (Fig. S7). However, notothenioid diversification rates in the Pleistocene still appear high in comparison, which may mostly be driven by the rapid radiation of Artedidraconidae within the Antarctic clade (see Near *et al*., 2012). Our time calibration indicates a very young age of Artedidraconidae (mean: 1.2 Ma, 95% HPD: 2.2–0.6 Ma), which suggests that repeated habitat fragmentation during glacial cycles of the Pleistocene may have acted as a diversity pump (Clark & Crame, 2010) in this high Antarctic family. However, phylogeographic analyses based on a more extensive taxon sampling of the 30 known artedidraconids will be required to corroborate this hypothesis.

### Disparity through time in Antarctic notothenioids

Our trait evolution simulations have shown that early bursts can be difficult to detect, especially when trait space is limited by hard or soft boundaries (Fig.4). Harmon *et al*. (2010) noted that early bursts are rare in comparative data sets, including classic examples of adaptive radiation. The authors fitted BM, OU and early burst models of body size and shape evolution to phylogenies of 49 animal clades and found that the early burst model received higher support than BM and OU models for only two of these clades. Results from our simulations suggest that if trait evolution in several of these clades was shaped by a combination of early burst and constrained evolution, testing for the two processes separately could easily fail to detect the early burst. With existing methods, trait likelihoods cannot be calculated for a model combining an early burst with soft trait space boundaries; however, such a model could potentially provide a better description of trait evolution in adaptive radiations if these would continue to evolve even after boundaries have been reached. It remains to be tested whether or not this is a commonly occurring process in adaptive radiation.

Even though the pattern of early burst could rapidly be blurred by constrained evolution, we observe a strongly negative MDI value, and therefore a signal for early burst, in CV1 of our geometric morphometric body shape data (Fig.5). Changes along CV1 affect mostly the shape and size of the snout. Long and pikelike snouts as well as shallower, more elongate bodies are associated with low CV1 values, and compressed and robust bodies and heads with very short snouts are characteristic for species with high CV1 values (Fig.2). These differences could reflect different feeding behaviours. Additional to the apparent correlation between mouth size and the size of prey that can be taken (i.e. larger mouth gapes allow the consumption of bigger prey items; Boubée & Ward, 1997; Adams & Huntingford, 2002), large and wide snouts and shallower bodies proved to be beneficial when preying on rapidly swimming, elusive target species using ram feeding, whereas short and robust snouts and bodies are better suited for suction feeding on more varied immobile prey (Webb, 1984; Norton & Brainerd, 1993; Huskey & Turingan, 2001). Differences in feeding and foraging modes may help to avoid interspecific competition and might therefore facilitate coexistence of sympatric species (Labropoulou & Eleftheriou, 1997). The highest CV1 values, and thus the most compressed snouts, are found in Trematomini and Artedidraconidae, whereas low CV1 values and elongated snouts and bodies are present in Channichthyidae, Cygnodraconinae and *G. acuticeps*. According to stomach content analyses, the diet of the latter three groups is dominated by swimming prey such as fish, krill and mysids, whereas Trematomini and Artedidraconidae feed on a varied diet that includes slowly moving organisms such as polychaetes, ophiuroids and echinoderms (see Table S3 in Rutschmann *et al*., 2011). As we observe little variance in CV1 values within individual notothenioid clades, we assume that diversification along CV1 may have driven the notothenioid radiation before the divergence of these clades. According to our time-calibrated phylogeny based on the best-supported model combination, this initial phase of diversification would have lasted from 13.4 Ma to about 9–7 Ma (Fig.1a). Taken together, these findings point towards a scenario where early diversification along CV1 primarily led to two distinct groups according to (trophic) morphology: (i) one that today comprises Channichthyidae, Cygnodraconinae and *G. acuticeps*, characterized by large, elongated snouts used for ram feeding on elusive prey such as fish, krill and mysids and (ii) one comprising lineages leading to Trematomini and Artedidraconidae, characterized by short, robust snouts and heads used for suction feeding on largely immobile prey such as polychaetes, ophiuroids and echinoderms.

If diversification along CV1 is driven by morphological specializations related to trophic resource acquisition, it could be seen as the second stage of adaptive radiation, as envisioned by Streelman & Danley (2003). Alternatively, morphological changes along this axis could be regarded as *α* niche diversification that facilitates local-scale coexistence between closely related species in the model of adaptive radiation proposed by Ackerly *et al*. (2006). In their model, *α* niche specialization occurs primarily in the beginning of an adaptive radiation, whereas differentiation of the *β*-niche relating to macrohabitat continues throughout the radiation. In agreement with this model, early diversification in trophic morphology has been suggested also in other prominent examples of adaptive radiation, including Neotropical (López-Fernández *et al*., 2013) and African cichlid fishes (Muschick *et al*., 2014). Among the five traits investigated by us, buoyancy and temperature regime could be regarded as *β*-niches, as diversification in both traits directly affects the habitat of notothenioids in the water column and their distribution range. Observed MDI values for buoyancy and temperature are close to zero (buoyancy; mean of tree sample: −0.016) or clearly positive (temperature; mean of tree sample: 1.456), indicating ongoing diversification along both of these axes. However, given that trait space for buoyancy values is limited by a hard lower boundary at 0 (assuming that obtaining negative buoyancy would require fundamentally different selection pressures that are not present in notothenioids), BM may not be the best model for the evolution of this trait. Similarly, trait space for notothenioid habitat temperatures is obviously limited by the freezing temperature of sea water. As our simulations have shown that similar DTT trajectories can result from both early bursts with bounded trait space and from unconstrained constant trait evolution (Fig.4d,i), potential early bursts in either buoyancy or temperature regime would be difficult or impossible to detect with DTT trajectories and can therefore not be excluded in notothenioids.

## Conclusion

Despite the difficulties associated with sampling in their remote environment, Antarctic notothenioid fishes are rapidly becoming a well-investigated model for an adaptive radiation in the marine realm. They have been shown to fulfil all criteria for adaptive radiations outlined by Schluter (2000), and one of the criteria, the correlation of phenotype and environment, has been demonstrated for at least two phenotypes, freeze protection and buoyancy adaptations. Whether or not other predictions of adaptive radiation theory, such as early bursts in diversity and disparity or evolution in stages, are supported by the notothenioid radiation has so far remained unclear. We here found evidence for a diversification rate increase at or near the origin of Antarctic notothenioids that may have coincided with the evolution of AFGPs. We also identified an early burst in trophic morphology of Antarctic notothenioids, a trait that is known to drive diversification in some of the most prominent adaptive radiations.

Methodologically, our extensive comparison of models for Bayesian phylogenetic inference has demonstrated how divergence time estimates of rapidly diversifying clades depend strongly on the choice of models and that time lines based on any single model should therefore be taken with caution. The fact that more parameter-rich models, and in particular models with unlinked gene trees, were generally better supported than simple models suggests that future phylogenetic investigations of notothenioids should include multiple individuals per species to allow more reliable estimation of coalescent parameters in the multispecies coalescent approach of *BEAST, which could also lead to further improvements in the estimation of the time line of the notothenioid radiation.

## Supporting Information

**Text S1**Partitioning of molecular markers. **Text S2**Species tree reconstruction. **Text S3**Kendall-Moran estimates of diversication in simulated phylogenies. **Text S4**The semi-diversied sampling scheme. **Figure S1**Landmarks used for geometric morphometric analyses. **Figure S2**Habitat parameters extracted from the AquaMaps database. **Figure S3**Maximum likelihood phylogenies for mitochondrial and nuclear markers. **Figure S4**10% majority-rule consensus tree of MP-EST species trees. **Figure S5**BEAST reanalysis of the MP-EST species tree topology. **Figure S6**Lineages through time. **Figure S7**Kendall-Moran estimates of diversification rates in five time intervals. **Figure S8**MCC tree with node numbers. **Table S1**Gene information for sequence markers. **Table S2**Sequence accession numbers. **Table S3**Mean values for notothenioid characteristics. **Table S4**Model combinations used in BEAST analyses. **Table S5**Model support and tree characteristics for BEAST analyses of the combined marker set. **Table S6**Model support and tree characteristics for BEAST analyses of the mitochondrial marker set. **Table S7**Model support and tree characteristics for BEAST analyses of the nuclear marker set. **Table S8**Species richness used for diversification rate analysis with MEDUSA. **Table S9**Sea surface temperatures of notothenioid habitats. **Table S10**Support values and ages estimates for nodes of the MCC tree.
