# Supplementary material for: Diversity and disparity through time in the adaptive radiation of Antarctic notothenioid fishes
Source: J Evol Biol. 2015 Jan 30;28(2):376–94. doi: 10.1111/jeb.12570 (PMC4407914; doi:10.1111/jeb.12570)
Supplement: Supplementary file 1 — Text S1Partitioning of molecular markers. Text S2Species tree reconstruction. Text S3Kendall-Moran estimates of diversication in simulated phylogenies. Text S4The semi-diversied sampling scheme. Figure S1Landmarks used for geometric morphometric analyses. Figure S2Habitat parameters extracted from the AquaMaps database. Figure S3Maximum likelihood phylogenies for mitochondrial and nuclear markers. Figure S410% majority-rule consensus tree of MP-EST species trees. Figure S5BEAST reanalysis of the MP-EST species tree topology. Figure S6Lineages through time. Figure S7Kendall-Moran estimates of diversification rates in five time intervals. Figure S8MCC tree with node numbers. Table S1Gene information for sequence markers. Table S2Sequence accession numbers. Table S3Mean values for notothenioid characteristics. Table S4Model combinations used in BEAST analyses. Table S5Model support and tree characteristics for BEAST analyses of the combined marker set. Table S6Model support and tree characteristics for BEAST analyses of the mitochondrial marker set. Table S7Model support and tree characteristics for BEAST analyses of the nuclear marker set. Table S8Species richness used for diversification rate analysis with MEDUSA. Table S9Sea surface temperatures of notothenioid habitats. Table S10Support values and ages estimates for nodes of the MCC tree. [file jeb0028-0376-sd1.pdf]

Supporting Information

Diversity and disparity through time in the adaptive radiation of Antarctic notothenioid fishes

Marco Colombo,[1] Malte Damerau,[2] Reinhold Hanel,[2] Walter Salzburger,[1,3†] and Michael Matschiner[1,3†]

Addresses:

[1]Zoological Institute, University of Basel, Vesalgasse 1, CH-4051 Basel, Switzerland

[2]Thünen-Institute for Fisheries Ecology, Palmaille 9, 22767 Hamburg, Germany

[3]Centre for Ecological and Evolutionary Synthesis (CEES), Department of Biosciences, University of Oslo, Oslo, Norway

[†]Corresponding author: E-mail: walter.salzburger@unibas.ch; michaelmatschiner@mac.com

1 Supplementary Text

Supplementary Text 1: Partitioning of molecular markers.
We used the software Concaterpillar v.1.7.2 (Leigh *et al.* 2008) to test for discordant evolutionary histories of the markers included in our data set. Here, and for all other phylogenetic analyses, the four mitochondrial alignments were concatenated and considered as a single marker. The Concaterpillar analysis was performed with default settings and assuming a GTR model of evolution, the only model of nucleotide substitutions available in the software. For tree inference, Concaterpillar was set up to use RAxML v.7.2.8 (Stamatakis 2006).

We found no significant discordance among nuclear markers. However, different evolutionary histories were detected for the concatenated nuclear alignment and the combined mitochondrial marker set (likelihood ratio test based on non-parametric bootstrapping; P ¡ 0.001). For the two sets of concordant markers, and for all individual markers, we conducted separate analyses with the software PartitionFinder v.1.1.1 (Lanfear *et al.* 2012) in order to determine the best-fitting partitioning schemes according to the Bayesian Information Criterion (BIC). In each analysis, primary data blocks were defined within marker sets according to gene and codon position. We used PartitionFinders greedy algorithm and assumed unlinked branch lengths for individual partitions. In separate analyses, we allowed PartitionFinder to test all substitution models available in BEAST, or the HKY+Gamma model only.

Regardless of whether all substitution models available in BEAST, or only the HKY+Gamma model was tested, PartitionFinder always identified two partitions in both the concatenated mitochondrial marker set and the combined nuclear marker set, where the first partition always grouped all first and second codon positions, and the second partition included third codon positions and, for the nuclear marker set, the intronic marker s7. In all analyses of individual nuclear markers, however, PartitionFinder identified only a single partition combining all codon positions.

Thus, all BEAST analyses (see Supplementary Text 2) with linked gene trees were performed with four partitions: mtdna_cp12 for the first two codon positions of mitochondrial markers (2272 bp), mtdna_cp3 for the third codon position of mitochondrial markers (1136 bp), nuclear_cp12 for the first and second codon position of all nuclear markers (2912 bp), and nuclear_cp3 for the third position of nuclear markers as well as s7 (1964 bp). In these analyses, parameters of substitution and clock models were unlinked among partitions. For BEAST analyses with unlinked gene trees (i.e. the *BEAST approach), we allowed individual gene trees for each nuclear marker, and defined a single partition per nuclear marker, as suggested by the results of our PartitionFinder analyses. Thus, we used nine partitions for BEAST analyses with unlinked gene trees: mtdna_cp12 and mtdna_cp3 as above, plus myh6 (705 bp), PTCHD4 (702 bp), enc1 (801 bp), tbr1b (618 bp), rps7 (508 bp), zic1 (837 bp), and snx33 (705 bp).

Supplementary Text 2: Species tree reconstruction.
Bayesian species tree reconstructions were performed with BEAST v.2.1 (Bouckaert *et al.* 2014) under a wide range of models and with both linked and unlinked (i.e. the multi- species coalescent model of *BEAST; Heled & Drummond 2010) gene trees for individual markers. Within each

marker, clock and substitution models remained unlinked for partitions identified by PartitionFinder, a practice that has been shown to improve Bayesian phylogenetic inference (Ho & Lanfear 2010). All analyses used the birth-death tree model (Gernhard 2008) and the same user-specified starting tree based on the phylogeny of Near *et al.* (2012), but we tested both the strict molecular clock and the uncorrelated lognormal (UCLN) relaxed molecular clock (Drummond *et al.* 2006) in separate analyses. Further, two different substitution models were assumed for all partitions: the HKY model (Hasegawa *et al.* 1985), as well as the reversible-jump based (RB) substitution model implemented in the RB add-on for BEAST 2 (Bouckaert *et al.* 2013; Drummond & Bouckaert 2014). Both substitution models were used in combination with a gamma distribution of among-site rate variation. Finally, base frequencies were either empirically determined or estimated in separate analyses.

Due to the lack of a reliable notothenioid fossil record (see Eastman & Grande 1991, Balushkin 1994), clock models were calibrated using three secondary divergence age constraints: Following Rutschmann *et al.* (2011) and Near *et al.* (2012), we applied normal prior distributions to the age of the separation of Bovichtidae (mean: 71.4 million years ago (Ma), standard deviation: 9.0 million years (myr)), Pseudaphritidae (mean: 63.0 Ma, standard deviation: 8.4 myr), and Eleginopsidae (mean: 42.9 Ma, standard deviation: 6.9 myr), based on the higher-level phylogeny of Matschiner *et al.* (2011) that was calibrated with 10 non-notothenioid fossil and biogeographic constraints. Contrary to Rutschmann *et al.* (2011) and Near *et al.* (2012), we chose not to constrain the age of the initial divergence of the Antarctic clade, as we expected that the multi-species coalescent model of *BEAST may detect previously unrecognized incomplete lineage sorting in the early phase of the Antarctic diversification, and may thus support younger species tree divergences compared to the concatenated analyses of Matschiner *et al.* (2011), Rutschmann *et al.* (2011), and Near *et al.* (2012).

Support for each model combination was assessed a posteriori using the Akaike Information Criterion through Markov Chain Monte Carlo (AICM) analysis (Raftery *et al.* 2007) which has been shown to perform favourably compared to marginal likelihoods obtained with the harmonic mean estimator (Baele *et al.* 2012). AICM values were estimated from posterior likelihood distributions with BEAST v.1.8 (Drummond *et al.* 2012), as this option is not implemented in BEAST v.2.1. All model combinations used for the BEAST analyses, together with the resulting AICM values are listed in Supplementary Tables 4-7. For each model combination, between three and nine replicate analyses with a total of 0.3 billion (when gene trees were linked) or 1.5-18 billion (when gene trees were unlinked) MCMC generations were performed, and run convergence was evaluated with effective sample sizes (ESS) and by visual inspection of MCMC traces within and between run replicates, using Tracer v.1.5 (Rambaut & Drummond 2007).

Supplementary Text 3: Kendall-Moran estimates of diversification in simulated phylogenies.
Kendall-Moran estimates (Dornburg *et al.* 2008; Near *et al.* 2012) for five time intervals of the Late Miocene (Tortonian, 11.6-7.2 Ma, and Messinian, 7.2-5.3 Ma), Pliocene (Zanclean, 5.3-3.6 Ma, and Piacenzian, 3.6-2.6 Ma), and Pleistocene (2.6-0 Ma) were calculated for notothenioid phylogenies and compared to null distributions obtained from phylogenies simulated with homogeneous diver-

sification rates. Using a pure-birth (Yule) model, we simulated 1000 phylogenies with the same age, and conditioned on the same extant species richness of (i) the Antarctic clade, or (ii) all Notothenioidei. In both cases, distributions of simulated root ages directly reflected ages of these two groups in the posterior sample of 1000 trees resulting from the BEAST analysis with the combined data set and the best-supported model combination. Simulations were performed with speciation rates λ drawn from wide uniform distribution between (i) 0.1 and 0.45 per myr, or (ii) between 0.02 and 0.2 per myr, and only those trees were retained that resulted in exactly (i) 123 or (ii) 134 extant species. In both cases, simulations were repeated until a total of 1000 phylogenies were found fulfilling these criteria. In order to account for unobserved extinction, we also repreated all simulations with a birth-death model, using a fixed extinction rate μ of 0.2 per myr, and correspondingly higher speciation rates λ between (i) 0.3 and 0.65 per myr, or (ii) 0.22 and 0.4 per myr, to result in the same net diversification rates as in the above Yule models.

All simulated phylogenies were subsequently sampled to match the number of representatives of (i) the Antarctic clade (45 species), or (ii) all Notothenioidei (49 species) included in our data set. For each phylogeny, this was performed according to two different sampling schemes: a random sampling scheme and a previously undescribed sampling scheme that tends to retain more older nodes than strictly random sampling. The incentives behind this sampling scheme are similar to those of the "diversified sampling" scheme of Höhna *et al.* (2011), which choses tips of a phylogeny so that diversity is maximized, and as a result samples all nodes in a phylogeny between its root and the time point at which the number of lineages matches that of sampled tips. Höhna *et al.* (2011) found this sampling scheme to provide a better fit to most phylogenies, as systematists usually attempt to include early-diverging lineages in their taxon sets (Cusimano & Renner 2010). However, at the stage at which systematists compile their taxon sets, the relative ages of lineages may be poorly known, or older lineages may be rare and difficult to sample. Thus, most empirical phylogenies may be more bottom-heavy than randomly sampled phylogenies, but not as bottom-heavy as phylogenies sampled according to the diversified sampling scheme.

Our notothenioid phylogeny is likely to fit this pattern. Like previous authors (Matschiner *et al.* 2011; Rutschmann *et al.* 2011; Near *et al.* 2012), we deliberately departed from a random sampling scheme by including representatives of all major lineages, even if their extant diversity is low, as is the case of Eleginopidae and Pseudaphritidae. However, not all of the oldest lineages could be sampled, as for example samples of *Halaphritis* and *Gvozdarus* could not be obtained. Thus, our empirical taxon sampling is intermediate between random and strictly diversified sampling of Notothenioidei, and as a consequence, an intermediate "semi-diversified" sampling scheme is likely to provide the best fit to our phylogeny. While not exploring the mathematical properties of this semi-diversified sampling scheme in detail (as done by Höhna *et al.*, 2011, for the diversified sampling scheme), we describe an algorithm to apply this scheme in Supplementary Text 4.

For all empirical and simulated phylogenies, we calculated Kendall-Moran estimates of diversification rates in each of the five time intervals using $b = (n - m)/B$, where n and m are the number of species extant at the beginning and end of the time interval, and B is the sum of all branch lengths within this interval (Becerra 2005). Densities of interval-specific diversification rate estimates in empirical and simulated phylogenies, as well as point estimates for the MCC tree and the

tree resulting from rerunning the best-supported model in BEAST with the topological constraint of the MP-EST species tree, are shown in Supplementary Figure 7.

Supplementary Text 4: The semi-diversified sampling scheme.
Let n be the number of extant species, m be the number of sampled extant species, and t_{root} the root age of a reconstructed tree. Then the number of sampled nodes is $m-1$, and if the tree was fully sampled ($m = n$), it would be $n-1$. In the diversified sampling scheme of Höhna *et al.* (2011), m species are sampled to maximize phylogenetic diversity, so that precisely the oldest $m-1$ nodes are present in the sampled tree. Thus, in this model, the probability p that a node is included in the sampled tree is 1 for the $m-1$ oldest nodes, and 0 for the $n-m$ younger nodes. However, for reasons explained in Supplementary Text 3, it is common that in empirical phylogenies, the realized sampling differs from the diversified sampling scheme so that some of the oldest $m-1$ nodes are missing, but some of the youngest $n-m$ nodes are present in the sampled tree. Among the oldest $m-1$ nodes, the younger ones are more likely to be missing, whereas among the youngest $n-m$ nodes, the older ones are more likely to be included. The probability that nodes are included in the sampled tree may thus be assumed to increase continuously with node age. Furthermore, the sampling probability of nodes with age $t_{node} = 0$ is 0, and for simplicity, we may assume that nodes with age t_{root} (the root only) are sampled with probability 1.

Thus, we here define the semi-diversified sampling scheme so that nodes are selected at random with uniform probability, and once selected, they are chosen to be sampled with acceptance probability $p_a(t) = \frac{t_{node}}{t_{root}}$. If a node is chosen to be sampled, one extant species is sampled randomly from the extant descendents of both sides of this node, so that the selected node necessarily appears in the sampled tree. This process is repeated until m extant taxa have been sampled. If a selected node is already present in the sampled tree (i.e. both of its descendent lineages are already represented in the list of sampled species), a new node is selected at random. In this model, the root node is sampled with probability $p_a = 1$ once it is selected, and it is automatically included in the sampled tree if the next-oldest nodes in both of its descendent lineages are sampled. However, this still leaves a small probability that it is not sampled in the case that it has not been selected before m extant species are sampled and if the next-oldest nodes in at least one of its descendent lineages is not sampled. Thus, for convenience, we may want to ensure that the root is included in the sampled tree. We can do this by sampling at random one extant species from each side of the root as the very first step of this process (if $m > 1$).

The effective sampling probability $p_s(t)$ that a node of age t is present in the sampled tree is different from the acceptance probability $p_a(t)$ for several reasons: First, the process is repeated multiple times until m extant species are sampled, so that nodes that were not sampled previously, can be selected again, and are then again sampled with probability $p_a(t)$. Second, even nodes that are not sampled directly can be included in the sampled tree if the next-oldest nodes in both of their descendent lineages are sampled. This will lead to an increase of the effective sampling probability in older nodes (only if $m > 2$). The effective sampling probability thus depends on the probability that a node is selected (however, this probability is here assumed uniform), on the acceptance probability $p_a(t)$ that a node is sampled once it is selected, and on the probability that the next-oldest nodes in

both descending lineages are chosen. The effective sampling probability further depends on m and n, because the process is repeated until m extant species are sampled. Thus, the effective sampling probability of a single node is also influenced by the probabilities of all other nodes, because if the other nodes' probabilities are low, the process will have to be repeated more often before m extant species are sampled. This means that a node's effective sampling probability is dependent not only on its own age, but also on the ages of all other nodes, and thus on the node age density. In a reconstructed continuous-rate birth-death process, conditioned on root age t_{root} and extant number of species n, this density is known from Gernhard (2008) and depends on speciation rate λ and extinction rate μ. Thus, calculation of the effective sampling probability may in principle be possible, but is not required in order to apply the semi-diversified sampling scheme, as long as we know that $p_s(t)$ has the desirable properties of, (i) $p_s(t_{root}) = 1$, (ii) $\int_0^{t_{root}} p_s(t) = m$, and (iii) continuous increase with t for $0 \leq t < t_{root}$. Properties i and ii are guaranteed, as (i) descendents from both sides of the root are sampled as a first step, and (ii) nodes can be sampled at most once, but the process is repeated until exactly m extant species are sampled. Without a proof, property iii is also assumed to be fulfilled, as $p_a(t)$ is continuously increasing with t for $0 \leq t < t_{root}$ and older nodes tend to have older descendants, which in turn increases the probability that these are accepted for sampling.

2 Supplementary Figures

Supplementary Figure 1: Landmarks used for geometric morphometric analyses.
Eighteen landmark points were chosen to quantify notothenioid body shape variation. Landmark points 1-17 are homologous to those used in Muschick *et al.* (2012, Figure S5).

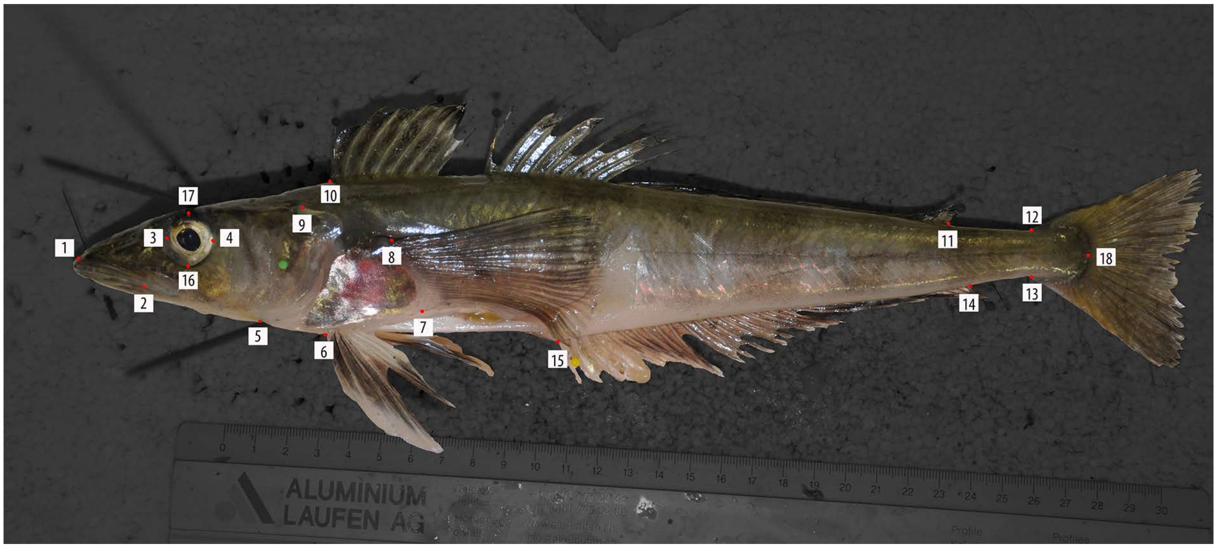

Supplementary Figure 2: Habitat parameters extracted from the AquaMaps database. For each species, black dots indicate means of observed trait values, and gray bars represent parameter range.

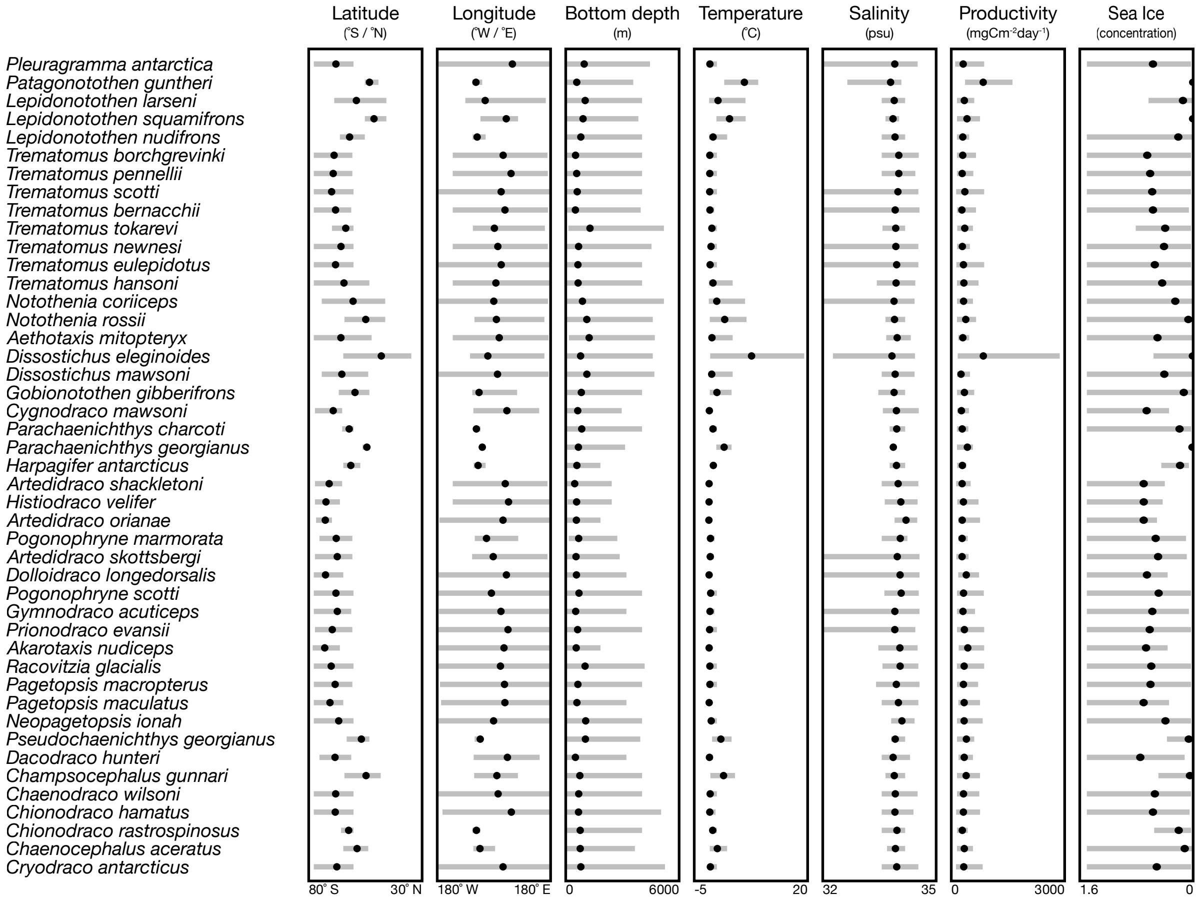

Supplementary Figure 3: Maximum likelihood phylogenies for mitochondrial and nuclear markers.
Phylogenies produced with RAxML for the two sets of concordant markers identified with Concaterpillar. The maximum likelihood tree for the concatenated mitochondrial marker set is shown at left, the tree based on concatenated nuclear markers is at right.

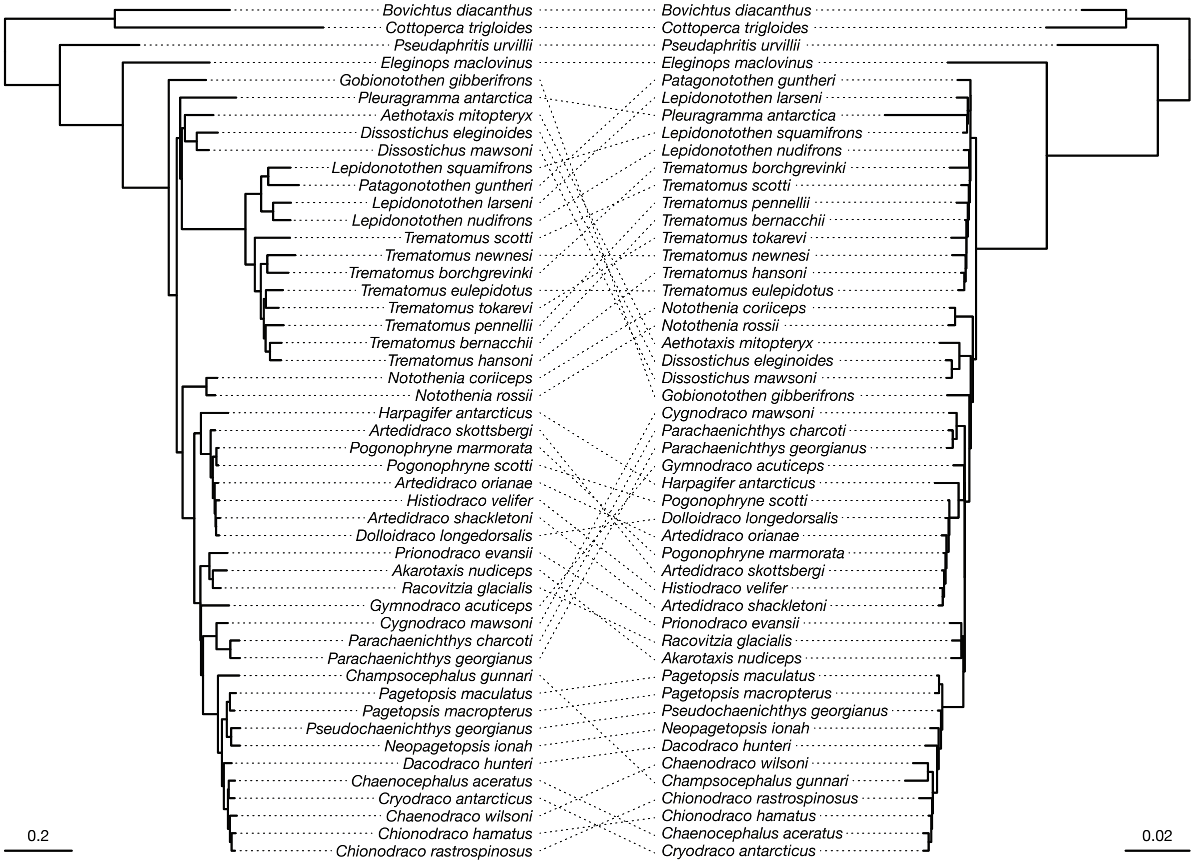

Supplementary Figure 4: 10% majority-rule consensus tree of MP-EST species trees.
Node labels indicate the number of bootstrap replicate MP-EST species trees supporting this node.

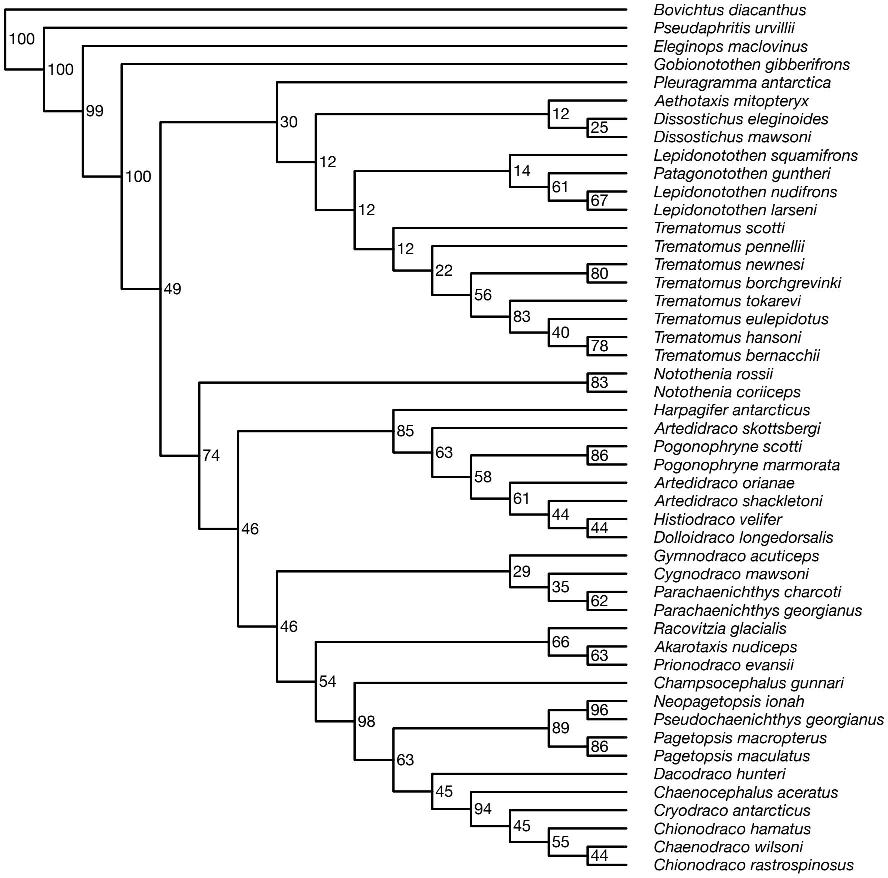

Supplementary Figure 5: BEAST reanalysis of the MP-EST species tree topology.
A) Phylogeny resulting from rerunning the BEAST analysis with the best-supported model combination, constrained to the topology of the MP-EST species tree (see Supplementary Figure 4). B) Comparison of posterior distributions resulting from the topologically unconstrained and constrained BEAST analyses of the combined data set, with the best-supported model combination according to AICM (models 12 and 12* in Supplementary Table 5).

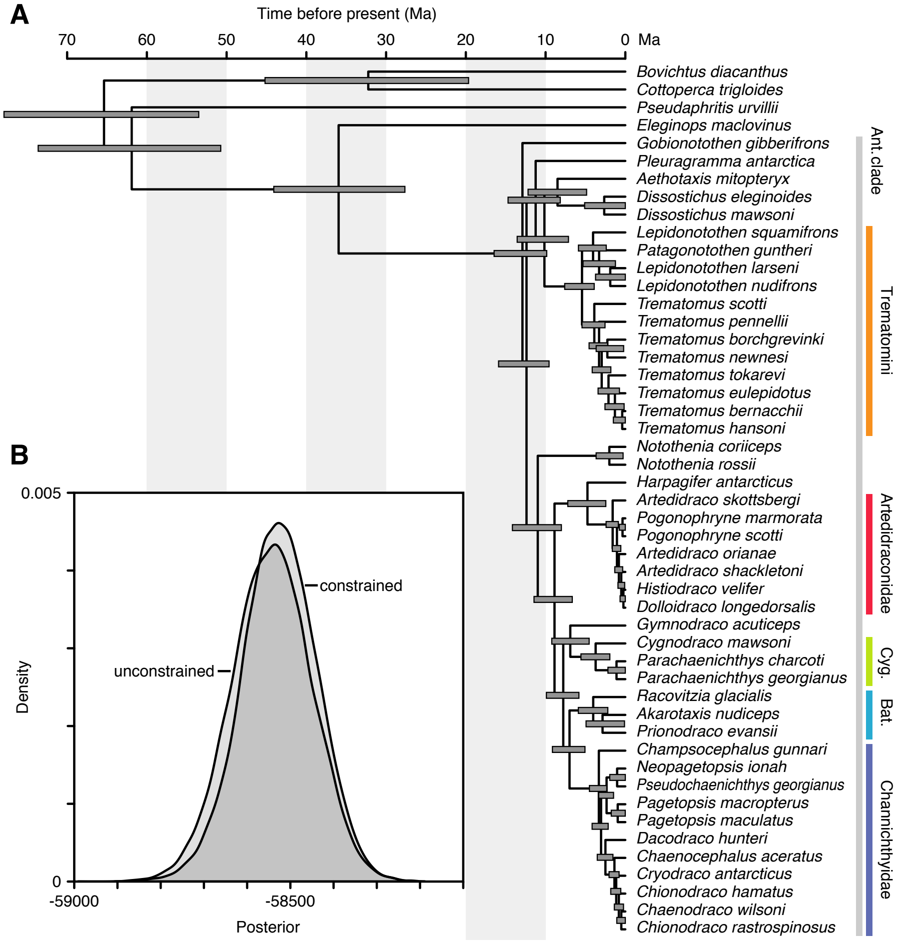

Supplementary Figure 6: Lineages through time.
Accumulation of lineages over time in time-calibrated phylogenies resulting from the BEAST analysis with the best-supported model combination. Gray lines represent number of lineages in 1000 posterior trees, the orange line shows the number of lineages in the MCC tree resulting from the same analysis. The blue line is based on mean age estimates of the BEAST analysis using the topological constraint of the MP-EST species tree.

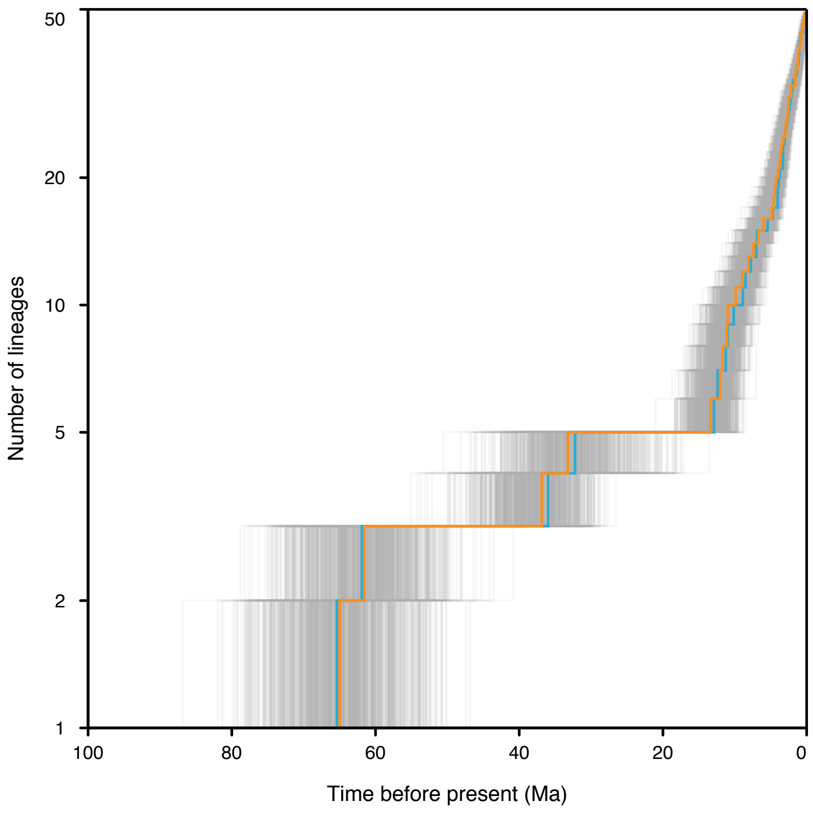

Supplementary Figure 7: Kendall-Moran estimates of diversification rates in five time intervals. Density distributions of Kendall-Moran diversification rate estimates in the posterior sample of 1000 trees resulting from the BEAST analysis of the combined data set and with the best-supported model combination (dashed line), mean values of these distributions (vertical black line), and rate estimates for the MCC tree (orange line) and the reanalysed MP-EST tree (blue line). The density distributions of simulated phylogenies, sampled with random sampling and the semi-diversified sampling scheme (see Supplementary Text 4), are shown as light gray shapes and dark gray lines, respectively. In A)-E) and K)-O), empirical phylogenies were trimmed to include only representatives of the Antarctic clade, and simulated phylogenies were conditioned on the age and species richness of this clade. In F)-J) and P)-T), the full empirical phylogenies were used, and simulated phylogenies were conditioned accordingly. A)-J) and K)-T) differ regarding the model used for simulated phylogenies (Yule or birth-death), but densities of diversification rates of empirical phylogenies are identical between these two sets. Orange, blue, and black asterisks indicate that rate estimates for the MCC tree, the reanalysed MP-EST tree, or mean rate estimates for the sample of 1000 trees, respectively, are larger than the 95% (*), 99% (**), or 99.9% (***) quantile of rates found in simulated phylogenies, after application of semi-diversified sampling.

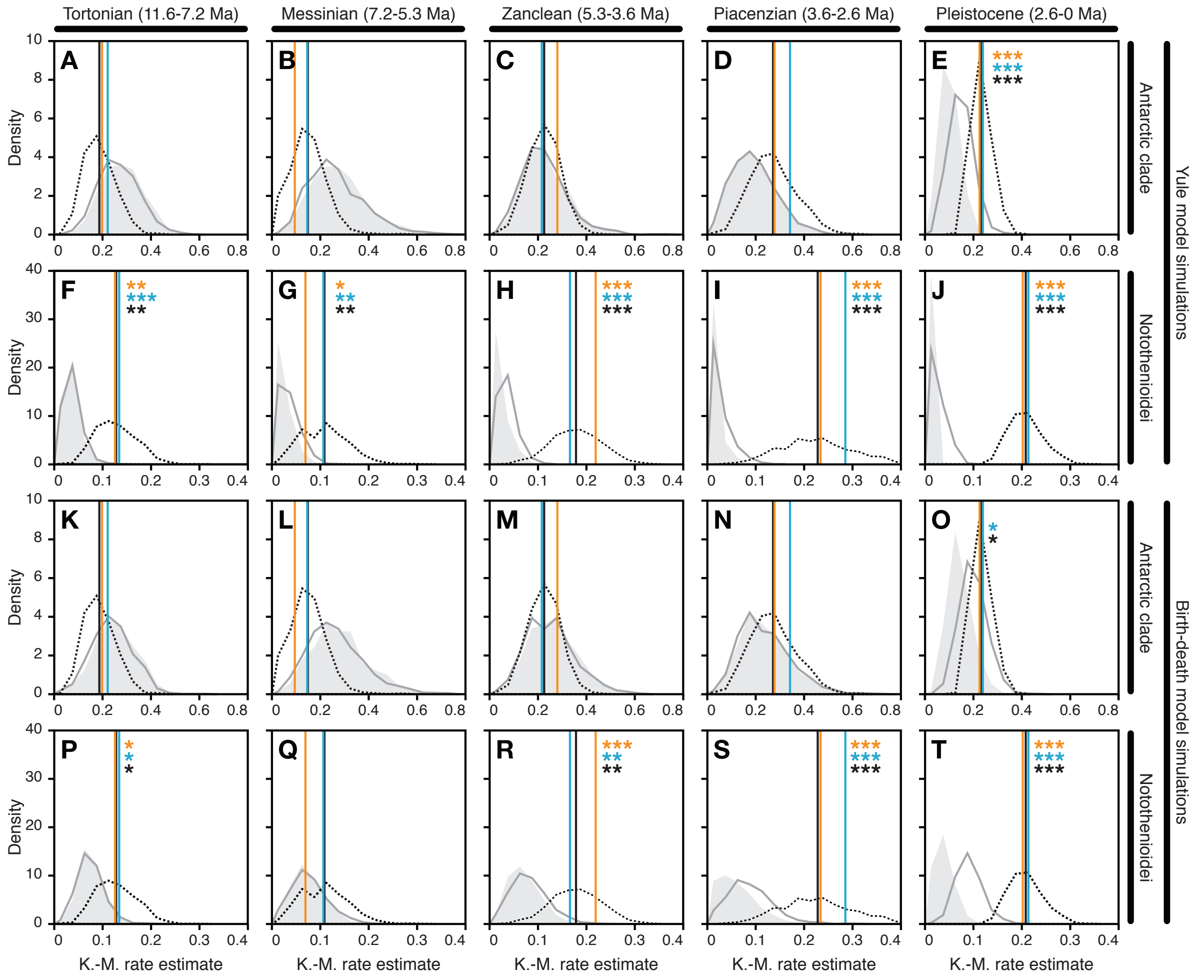

Supplementary Figure 8: MCC tree with node numbers.
The phylogeny is identical to the tree shown in Fig. 1, but with labels indicating node numbers. See Supplementary Table 10 for BPP values and age estimates for each node.

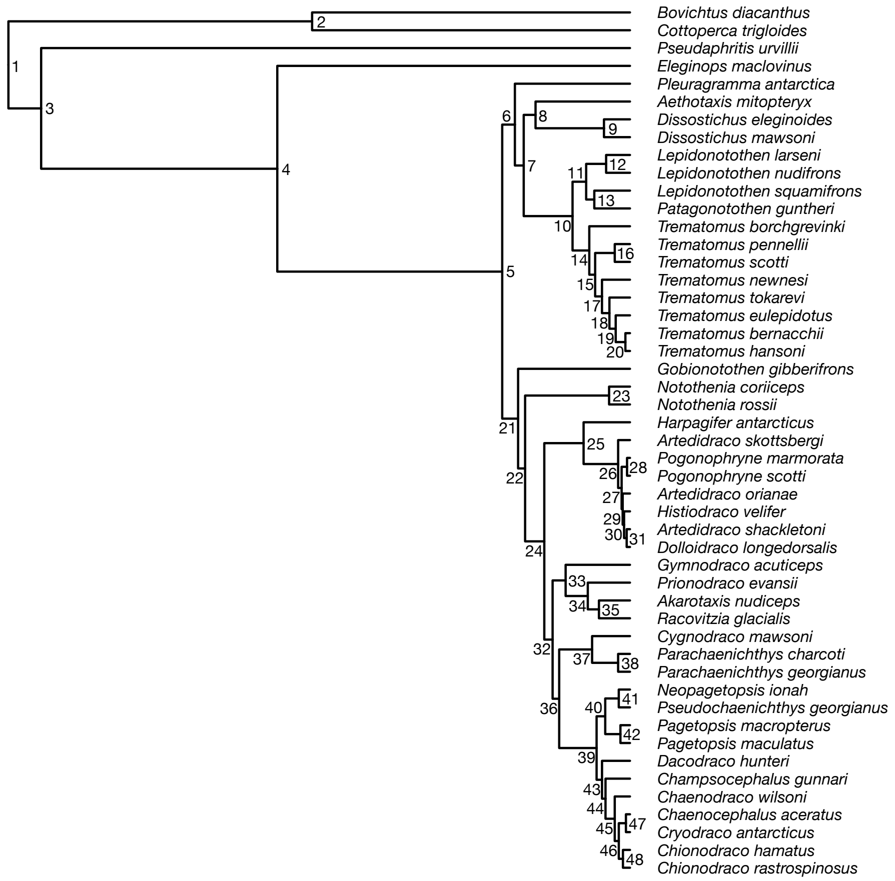

3 Supplementary Tables

Supplementary Table 1: Gene information for sequence markers.
Symbol and name refer to the official gene symbol and name in zebrafish, as listed in the ZFIN database (Sprague *et al.* 2006). Synonyms are given as used in the reference. The location column specifies the location of the marker sequence within this gene, and length refers to the length of each marker's sequence alignment. See references for primer information.

Symbol	Name	Synonym	Location	Length	Reference
mt-co1	cytochrome c oxidase I, mitochondrial	COI	Exon 1	651 bp	Ratnasingham & Hebert (2007)
mt-cyb	cytochrome b, mitochondrial	cyt b	Exon 1	1080 bp	Matschiner *et al.* (2011)
mt-nd2	NADH dehydrogenase 2, mitochondrial	nd2	Exon 1	1044 bp	Near *et al.* (2012)
mt-nd4	NADH dehydrogenase 4, mitochondrial	nd4	Exon 1	633 bp	Matschiner *et al.* (2011)
enc1	ectodermal-neural cortex 1	ENC1	Exon 3	801 bp	Li *et al.* (2007)
myh6	myosin, heavy polypeptide 6, cardiac muscle, alpha	-	Exon 1	705 bp	Li *et al.* (2007)
PTCHD4	patched domain containing 4	Ptr	Exon 3	702 bp	Li *et al.* (2007)
rps7	ribosomal protein S7	S7	Intron 1	508 bp	Chow & Hazama (1998)
snx33	sorting nexin 33	SH3PX3	Exon 1	705 bp	Li *et al.* (2007)
tbr1b	T-box, brain, 1b	tbr1	Exon 6	618 bp	Li *et al.* (2007)
zic1	zic family member 1 (odd-paired homolog, Drosophila)	-	Exon 1	837 bp	Li *et al.* (2007)

Supplementary Table 2: Sequence accession numbers.
Sequences marked with * were produced for this study, those marked with † were taken from BOLD, all other sequences are from Genbank.
See separate file.

Supplementary Table 3 (next page): Mean values for notothenioid characteristics.
Per species, sample size and the mean values for the first two canonical variates of body shape, as well as body size (in cm), buoyancy and sea surface temperature (in °C) are listed. For body size, we used maximum terminal lengths (TL) reported by Gon & Heemstra (1990). For species, for which maximum lengths were given as standard lengths (SL) in Gon & Heemstra (1990), we transformed these values to TL based on per-species mean TL/SL ratios empirically determined from specimens included in our data set.

Supplementary Table 3 (continued)

Species	#	Shape CV1	Shape CV2	Body size	Buoyancy	Temperature
Akarotaxis nudiceps	2	-4.223	15.546	15.0	3.84	-1.71
Artedidraco shackletoni	2	4.547	-2.552	14.6		-1.72
Artedidraco skottsbergi	5	6.472	-2.879	10.6	5.40	-1.47
Chaenocephalus aceratus	39	-8.931	-0.842	75.0	3.19	0.10
Champsocephalus gunnari	41	-4.977	-2.950	66.0	2.90	1.47
Chaenodraco wilsoni	34	-5.087	-2.507	43.0	3.08	-1.49
Chionodraco hamatus	2	-11.039	-0.925	49.0		-1.57
Chionodraco rastrospinosus	28	-8.541	-2.029	52.0	2.72	-0.93
Cryodraco antarcticus	43	-9.503	-2.716	57.0	2.53	-1.44
Cygnodraco mawsoni	4	-10.255	4.462	44.9		-1.67
Dacodraco hunteri	2	-10.881	0.595	29.0	1.41	-1.65
Dissostichus eleginoides	14	0.990	4.483	215.0		7.64
Dissostichus mawsoni	25	0.027	4.518	175.0	0.00	-1.15
Dolloidraco longedorsalis	1	7.689	-3.427	13.7	4.49	-1.72
Gobionotothen gibberifrons	44	2.344	0.867	55.0	4.27	0.00
Gymnodraco acuticeps	2	-10.811	22.875	38.1	3.38	-1.52
Harpagifer antarcticus	2	3.315	6.732	11.4	5.99	-0.79
Histiodraco velifer	1	4.712	-2.850	19.2		-1.73
Lepidonotothen larseni	42	5.501	-1.743	24.0	4.22	0.24
Lepidonotothen nudifrons	31	6.648	-5.438	19.0	4.46	-0.85
Lepidonotothen squamifrons	61	6.771	-0.187	55.0	3.20	2.78
Neopagetopsis ionah	11	-7.946	-1.795	56.0	1.28	-1.26
Notothenia coriiceps	27	4.650	1.658	62.0	3.67	-0.04
Notothenia rossii	30	4.825	1.486	92.0	3.55	1.72
Pagetopsis macropterus	7	-6.845	-3.709	33.0	2.38	-1.55
Pagetopsis maculatus	4	-6.092	-0.784	25.0	3.11	-1.71
Parachaenichthys charcoti	1	-10.436	12.935	46.7	4.39	-0.87
Parachaenichthys georgianus	11	-9.633	11.134	65.1		1.59
Patagonotothen guntheri	11	4.412	-0.384	23.0		6.06
Pleuragramma antarctica	25	0.837	-0.041	25.0	0.34	-1.55
Pogonophryne marmorata	2	0.732	13.787	21.0	3.81	-1.42
Pogonophryne scotti	1	1.341	10.869	31.0	3.80	-1.38
Prionodraco evansii	5	2.946	29.614	17.0	4.21	-1.61
Pseudochaenichthys georgianus	31	-9.140	1.264	60.0	1.96	0.87
Racovitzia glacialis	1	-3.377	-2.505	27.4	4.08	-1.55
Trematomus bernacchii	7	5.159	-0.157	35.0	3.52	-1.54
Trematomus eulepidotus	44	6.549	-0.735	34.0	3.41	-1.50
Trematomus hansoni	19	4.621	-0.561	41.0	3.12	-0.86
Trematomus newnesi	8	6.290	-0.765	20.0	3.76	-1.30
Trematomus pennellii	11	5.817	-2.001	24.0	3.09	-1.57
Trematomus scotti	21	6.343	2.060	16.0	4.14	-1.59
Trematomus tokarevi	1	5.600	5.435	22.4	2.77	-1.11

Supplementary Table 4: Model combinations used in BEAST analyses.
Twelve combinations of gene tree linkage, substitution model, base frequency setting, and clock model are listed. Supplementary Tables 4-6 refer to these model combinations.

Model	Gene trees	Substitution model	Frequencies	Clock model
1	linked	HKY+Gamma	empirical	strict
2	linked	HKY+Gamma	estimated	strict
3	linked	HKY+Gamma	estimated	UCLN
4	linked	RB+Gamma	empirical	strict
5	linked	RB+Gamma	estimated	strict
6	linked	RB+Gamma	estimated	UCLN
7	unlinked	HKY+Gamma	empirical	strict
8	unlinked	HKY+Gamma	estimated	strict
9	unlinked	HKY+Gamma	estimated	UCLN
10	unlinked	RB+Gamma	empirical	strict
11	unlinked	RB+Gamma	estimated	strict
12	unlinked	RB+Gamma	estimated	UCLN

Supplementary Table 5: Model support and tree characteristics for BEAST analyses of the combined marker set.
For each model combination, AICM values, Akaike weights, the number of parameters, the mean BPP, and the mean TMRCA of the diversification of Antarctic notothenioids are listed. Model combination numbers refer to those listed in Supplementary Table 4. Model 12* is identical to model 12, but with the species tree topology constrained to that of the species tree obtained with MP-EST.

Model	AICM	ΔAICM	Akaike weight	Parameters	Mean BPP	TMRCA Antarctic Clade
1	117355.4	1242.0	0.00000	15	0.967	22.36
2	117076.1	962.6	0.00000	19	0.965	22.54
3	116288.8	175.3	0.00000	27	0.964	22.37
4	116952.5	839.1	0.00000	19	0.967	22.32
5	116965.0	851.6	0.00000	23	0.968	22.35
6	116161.9	48.4	0.00000	31	0.971	22.35
7	116963.1	849.7	0.00000	40	0.663	18.67
8	116711.3	597.8	0.00000	49	0.670	18.30
9	116181.2	67.8	0.00000	67	0.684	13.31
10	116575.8	462.3	0.00000	49	0.668	17.62
11	116614.2	500.7	0.00000	58	0.670	17.69
12	116113.4	0.0	0.99956	76	0.684	13.35
12*	116128.9	15.5	0.00044	76	1.000	12.90

Supplementary Table 6: Model support and tree characteristics for BEAST analyses of the mitochondrial marker set.
For each model combination, AICM values, Akaike weights, the number of parameters, the mean BPP, and the mean TMRCA of the diversification of Antarctic notothenioids are listed. Model combination numbers refer to those listed in Supplementary Table 4.

Model	AICM	ΔAICM	Akaike weight	Parameters	Mean BPP	TMRCA Antarctic Clade
1	83604.3	686.5	0.00000	9	0.971	28.20
2	83341.0	423.2	0.00000	11	0.978	28.49
3	83065.2	147.3	0.00000	15	0.957	26.31
4	83243.8	326.0	0.00000	11	0.974	28.19
5	83254.9	337.0	0.00000	13	0.975	28.17
6	82929.3	11.5	0.00321	17	0.964	26.08
7	83607.6	689.8	0.00000	12	0.435	25.54
8	83342.4	424.6	0.00000	14	0.451	25.99
9	83061.7	143.9	0.00000	18	0.397	23.97
10	83243.2	325.4	0.00000	14	0.459	25.81
11	83254.2	336.4	0.00000	16	0.471	25.99
12	82917.8	0.0	0.99679	20	0.443	23.46

Supplementary Table 7: Model support and tree characteristics for BEAST analyses of the nuclear marker set.
For each model combination, AICM values, Akaike weights, the number of parameters, the mean BPP, and the mean TMRCA of the diversification of the Antarctic Clade are listed. Model combination numbers refer to those listed in Supplementary Table 4.

Model	AICM	ΔAICM	Akaike weight	Parameters	Mean BPP	TMRCA Antarctic Clade
1	33313.2	266.6	0.00000	9	0.860	18.92
2	33297.8	251.2	0.00000	11	0.856	19.01
3	33064.7	18.1	0.00012	15	0.808	17.51
4	33273.6	227.0	0.00000	11	0.847	19.09
5	33275.1	228.6	0.00000	13	0.847	19.10
6	33046.6	0.0	0.99988	17	0.794	17.76
7	33270.0	223.4	0.00000	33	0.603	15.28
8	33299.4	252.8	0.00000	40	0.611	15.20
9	33163.5	116.9	0.00000	54	0.570	10.90
10	33242.1	195.5	0.00000	40	0.607	14.92
11	33260.3	213.8	0.00000	47	0.609	14.90
12	33142.5	95.9	0.00000	61	0.582	11.18

Supplementary Table 8: Species richness used for diversification rate analysis with MEDUSA. Number of extant species according to Eastman & Eakin (2000, updated Table 1, version dating from 10 July 2013, available at http://www.oucom.ohiou.edu/dbms-eastman/). Clades were chosen so that the entire extant diversity of the notothenioid suborder could be assigned to them, with two exceptions: we exclude the monotypic genus *Gvozdarus*, which has been provisionally assigned to the non-monophyletic family Nototheniidae (Dettai *et al.* 2012), but is known from only two specimens (Fenaughty *et al.* 2008), of which no molecular sequence data has been produced. Similarly, the monotypic genus *Halaphritis* is known from only three specimens collected off the coast of Tasmania, and attempts to extract DNA from these samples have remained unsuccessful (Last *et al.* 2002). This species has been provisionally assigned to the family Bovichtidae, but shares its biogeographic distribution and morphological characteristics with *Pseudaphritis*, so that its phylogenetic placement remains questionable (Last *et al.* 2002). Thus, we here ignore both *Gvozdarus* and *Halaphritis*.

Family	Clade	Richness
Bovichtidae	*Bovichtus*	7
Bovichtidae	*Cottoperca*	1
Pseudaphritidae	Pseudaphritidae	1
Eleginopidae	Eleginopidae	1
Nototheniidae	*Aethotaxis*	1
Nototheniidae	*Pleuragramma*	1
Nototheniidae	*Dissostichus*	2
Nototheniidae	*Gobionotothen*	4
Nototheniidae	*Notothenia+Paranotothenia*	7
Nototheniidae	Trematominae	34
Harpagiferidae	Harpagiferidae	11
Artedidraconidae	Artedidraconidae	30
Bathydraconidae	Bathydraconinae[1]	9
Bathydraconidae	Cygnodraconinae[1]	4
Bathydraconidae	Gymnodraconinae[1]	3
Channichthyidae	*Chaenocephalus*	1
Channichthyidae	*Chaenodraco*	1
Channichthyidae	*Champsocephalus*	2
Channichthyidae	*Chionodraco*	3
Channichthyidae	*Cryodraco+Channichthys+Chionobathyscus*[2]	4
Channichthyidae	*Dacodraco*	1
Channichthyidae	*Neopagetopsis*	1
Channichthyidae	*Pagetopsis*	2
Channichthyidae	*Pseudochaenichthys*	1
Sum		132

[1]We here follow the subfamilial classification of Derome *et al.* (2002), but also consider *Akarotaxis* and *Vomeridens* to be part of Bathydraconinae, according to the molecular phylogenies of Near *et al.* (2012) and Dettai *et al.* (2012).

[2]Both *Channichthys* and *Chionobathyscus* are missing in our data set, however the phylogenetic analyses of Near *et al.* (2012) suggest that these two genera are most closely related to *Cryodraco*.

Supplementary Table 9: Sea surface temperatures of notothenioid habitats.
For each species, latitude and longitude of geographic grid cell centers are listed, in which this species is known to occur, as well as sea surface temperature extracted for these grid cells from the Optimum Interpolation Sea Surface Temperature atlas.
See separate file.

Supplementary Table 10: Support values and ages estimates for nodes of the MCC tree.
BPP values, mean divergence dates estimates, and 95% HPD intervals for all nodes of the MCC tree resulting from the BEAST analysis of the combined marker set with the best-supported model combination. Bootstrap (BS) values show the number of bootstrapped replicates of the MP-EST species tree analysis that support this node. Node labels refer to those given in Supplementary Figure 8.

Node	BPP	Mean age	95% HPD	BS	Node	BPP	Mean age	95% HPD	BS
1	1.00	64.96	77.24-53.15	100	25	1.00	4.85	7.31-2.49	85
2	1.00	33.22	46.16-20.35	-	26	1.00	1.24	2.19-0.56	63
3	1.00	61.54	72.77-50.15	100	27	0.55	0.87	1.39-0.46	58
4	1.00	36.86	45.40-28.89	99	28	0.46	0.31	0.70-0.00	86
5	1.00	13.35	17.12-9.98	100	29	0.14	0.77	1.28-0.30	61
6	0.26	12.03	16.00-8.66	30	30	0.22	0.62	1.10-0.13	44
7	0.16	11.11	14.89-7.46	12	31	0.18	0.35	0.83-0.00	11
8	0.73	9.87	14.31-5.11	12	32	0.78	8.09	10.35-6.09	46
9	1.00	2.72	5.47-0.00	25	33	0.42	6.73	9.24-4.30	1
10	1.00	6.00	8.11-4.20	12	34	1.00	4.41	6.46-2.56	66
11	0.74	4.58	6.79-2.57	14	35	0.64	3.25	5.38-0.78	15
12	0.80	2.50	4.55-0.00	67	36	0.37	7.40	9.56-5.29	2
13	0.60	3.73	6.12-0.66	10	37	1.00	3.96	5.87-2.10	35
14	0.98	4.26	5.78-2.86	12	38	1.00	1.24	2.41-0.00	62
15	0.50	3.64	5.07-2.28	0	39	1.00	3.49	4.57-2.44	98
16	0.46	1.59	3.44-0.00	4	40	0.81	2.59	3.63-1.60	89
17	0.31	2.93	4.35-1.46	2	41	0.98	1.17	2.19-0.00	96
18	0.49	2.15	3.61-0.55	83	42	1.00	1.00	1.89-0.00	86
19	0.34	1.49	2.97-0.16	40	43	0.73	2.92	3.98-2.00	1
20	0.57	0.48	1.96-0.00	78	44	0.25	2.56	3.56-1.61	17
21	0.48	11.69	15.30-8.64	8	45	0.99	1.59	2.30-0.98	94
22	0.40	10.96	14.30-8.19	74	46	0.40	1.17	1.88-0.51	3
23	1.00	2.18	3.81-0.31	83	47	0.74	0.43	1.20-0.00	2
24	1.00	8.96	11.53-6.72	46	48	0.70	0.74	1.36-0.00	16

References

Baele G, Lemey P, Bedford T *et al.* (2012) Improving the accuracy of demographic and molecular clock model comparison while accommodating phylogenetic uncertainty. *Mol Biol Evol*, **29**, 2157–2167.

Balushkin AV (1994) Fossil notothenioid, and not gadiform, fish *Proeleginops grandeastmanorum* gen. sp. nov. (Perciformes, Notothenioidei, Eleginopidae) from the late Eocene found in Seymour Island (Antarctica). *Voprosy Ikhtiologii*, **34**, 298–307.

Becerra JX (2005) Timing the origin and expansion of the Mexican tropical dry forest. *Proc Natl Acad Sci*, **102**, 10919–10923.

Bouckaert R, Alvarado-Mora MV, Rebello Pinho JR (2013) Evolutionary rates and HBV: issues of rate estimation with Bayesian molecular methods. *Antivir Ther*, **18**, 497–503.

Bouckaert R, Heled J, Kühnert D *et al.* (2014) BEAST 2: a software platform for Bayesian evolutionary analysis. *PLoS Computational Biology*, **10**, e1003537.

Chow S, Hazama K (1998) Universal PCR primers for S7 ribosomal protein gene introns in fish. *Mol Ecol*, **7**, 1255–1256.

Cusimano N, Renner SS (2010) Slowdowns in diversification rates from real phylogenies may not be real. *Syst Biol*, **59**, 458–464.

Derome N, Chen WJ, Dettaï A, Bonillo C, Lecointre G (2002) Phylogeny of Antarctic dragonfishes (Bathydraconidae, Notothenioidei, Teleostei) and related families based on their anatomy and two mitochondrial genes. *Mol Phylogenet Evol*, **24**, 139–152.

Dettai A, Berkani M, Lautrédou AC *et al.* (2012) Tracking the elusive monophyly of nototheniid fishes (Teleostei) with multiple mitochondrial and nuclear markers. *Mar Genomics*, **8**, 49–58.

Dornburg A, Santini F, Alfaro M (2008) The influence of model averaging on clade posteriors: an example using the triggerfishes (family Balistidae). *Syst Biol*, **57**, 905–919.

Drummond AJ, Bouckaert R (2014) *Bayesian Evolutionary Analysis with BEAST 2*. Cambridge University Press.

Drummond AJ, Ho SYW, Philips MJ, Rambaut A (2006) Relaxed phylogenetics and dating with confidence. *PLOS Biology*, **4**, e88.

Drummond AJ, Suchard MA, Xie D, Rambaut A (2012) Bayesian phylogenetics with BEAUti and the BEAST 1.7. *Mol Biol Evol*, **29**, 1969–1973.

Eastman JT, Eakin RR (2000) An updated species list for notothenioid fish (Perciformes; Notothenioidei), with comments on Antarctic species. *Arch Fish Mar Res*, **48**, 11–20.

Eastman JT, Grande L (1991) Late Eocene gadiform (Teleostei) skull from Seymour Island, Antarctic Peninsula. *Antarct Sci*, **3**, 87–95.

Fenaughty JM, Eastman JT, Sidell BD (2008) Biological implications of low condition factor "axe handle" specimens of the Antarctic toothfish, Dissostichus mawsoni, from the Ross Sea. *Antarct Sci*, **20**, 537–551.

Gernhard T (2008) The conditioned reconstructed process. *J Theor Biol*, **253**, 769–778.

Gon O, Heemstra PC (1990) *Fishes of the Southern Ocean.* J.L.B. Smith Institute of Ichthyology, Grahamstown, South Africa.

Hasegawa M, Kishino H, Yano Ta (1985) Dating of the human-ape splitting by a molecular clock of mitochondrial DNA. *Journal of Molecular Evolution*, **22**, 160–174.

Heled J, Drummond AJ (2010) Bayesian inference of species trees from multilocus data. *Mol Biol Evol*, **27**, 570–580.

Ho SYW, Lanfear R (2010) Improved characterisation of among-lineage rate variation in cetacean mitogenomes using codon-partitioned relaxed clocks. *Mitochondrial DNA*, **21**, 138–146.

Höhna S, Stadler T, Ronquist F, Britton T (2011) Inferring speciation and extinction rates under different sampling schemes. *Mol Biol Evol*, **28**, 2577–2589.

Lanfear R, Calcott B, Ho SYW, Guindon S (2012) PartitionFinder: combined selection of partitioning schemes and substitution models for phylogenetic analyses. *Mol Biol Evol*, **29**, 1695–1701.

Last PR, Balushkin AV, Hutchins JB (2002) *Halaphritis platycephala* (Notothenioidei: Bovichtidae): A new genus and species of temperate icefish from Southeastern Australia. *Copeia*, **2002**, 433–440.

Leigh JW, Susko E, Baumgartner M, Roger AJ (2008) Testing congruence in phylogenomic analysis. *Syst Biol*, **57**, 104–115.

Li C, Ortí G, Zhang G, Lu G (2007) A practical approach to phylogenomics: the phylogeny of ray-finned fish (Actinopterygii) as a case study. *BMC Evolutionary Biology*, **7**, 44.

Matschiner M, Hanel R, Salzburger W (2011) On the origin and trigger of the notothenioid adaptive radiation. *PLOS ONE*, **6**, e18911.

Muschick M, Indermaur A, Salzburger W (2012) Convergent evolution within an adaptive radiation of cichlid fishes. *Curr Biol*, **22**, 2362–2368.

Near TJ, Dornburg A, Kuhn KL *et al.* (2012) Ancient climate change, antifreeze, and the evolutionary diversification of Antarctic fishes. *Proc Natl Acad Sci*, **109**, 3434–3439.

Raftery AE, Newton M, Satagopan J, Krivitsky P (2007) Estimating the integrated likelihood via posterior simulation using the harmonic mean identity. In: *Bayesian Statistics* (eds. Bernardo JM, Bayarri MJ, Berger JO). Oxford University Press, Oxford.

Rambaut A, Drummond AJ (2007) Tracer v1.5.

Ratnasingham S, Hebert PDN (2007) BOLD: The Barcode of Life Data System (www.barcodinglife.org). *Molecular Ecology Notes*, **7**, 355–364.

Rutschmann S, Matschiner M, Damerau M *et al.* (2011) Parallel ecological diversification in Antarctic notothenioid fishes as evidence for adaptive radiation. *Mol Ecol*, **20**, 4707–4721.

Sprague J, Bayraktaroglu L, Clements D *et al.* (2006) The Zebrafish Information Network: the zebrafish model organism database. *Nucl Acids Res*, **34**, D581–5.

Stamatakis A (2006) RAxML-VI-HPC: maximum likelihood-based phylogenetic analyses with thousands of taxa and mixed models. *Bioinformatics*, **22**, 2688–2690.
